# Mega‐sized pericentromeric blocks of simple telomeric repeats and their variants reveal patterns of chromosome evolution in ancient Cycadales genomes

**DOI:** 10.1111/tpj.15969

**Published:** 2022-10-11

**Authors:** Radka Vozárová, Wencai Wang, Jana Lunerová, Fengqing Shao, Jaume Pellicer, Ilia J. Leitch, Andrew R. Leitch, Aleš Kovařík

**Affiliations:** ^1^ Department of Molecular Epigenetics Institute of Biophysics, Czech Academy of Sciences v.v.i., Královopolská 135 612 65 Brno Czech Republic; ^2^ Department of Experimental Biology, Faculty of Science Masaryk University 611 37 Brno Czech Republic; ^3^ Science and Technology Innovation Centre Guangzhou University of Chinese Medicine Guangzhou 510405 China; ^4^ Royal Botanic Gardens Kew, Richmond Surrey TW9 3AB UK; ^5^ Institut Botànic de Barcelona (IBB, CSIC‐Ajuntament de Barcelona) Passeig del Migdia sn 08038 Barcelona Spain; ^6^ School of Biological and Chemical Sciences Queen Mary University of London London E1 4NS UK

**Keywords:** telomeres, genome evolution, chromosome rearrangements, Cycadaceae, gymnosperms, centromeres, epigenetics

## Abstract

Simple telomeric repeats composed of six to seven iterating nucleotide units are important sequences typically found at the ends of chromosomes. Here we analyzed their abundance and homogeneity in 42 gymnosperm (29 newly sequenced), 29 angiosperm (one newly sequenced), and eight bryophytes using bioinformatics, conventional cytogenetic and molecular biology approaches to explore their diversity across land plants. We found more than 10 000‐fold variation in the amounts of telomeric repeats among the investigated taxa. Repeat abundance was positively correlated with increasing intragenomic sequence heterogeneity and occurrence at non‐telomeric positions, but there was no correlation with genome size. The highest abundance/heterogeneity was found in the gymnosperm genus *Cycas* (Cycadaceae), in which megabase‐sized blocks of telomeric repeats (i.e., billions of copies) were identified. Fluorescent *in situ* hybridization experiments using variant‐specific probes revealed canonical *Arabidopsis*‐type telomeric TTTAGGG repeats at chromosome ends, while pericentromeric blocks comprised at least four major telomeric variants with decreasing abundance: TTTAGGG>TTCAGGG >TTTAAGG>TTCAAGG. Such a diversity of repeats was not found in the sister cycad family Zamiaceae or in any other species analyzed. Using immunocytochemistry, we showed that the pericentromeric blocks of telomeric repeats overlapped with histone H3 serine 10 phosphorylation signals. We show that species of *Cycas* have amplified their telomeric repeats in centromeric and telomeric positions on telocentric chromosomes to extraordinary high levels. The ancestral chromosome number reconstruction suggests their occurrence is unlikely to be the product of ancient Robertsonian chromosome fusions. We speculate as to how the observed chromosome dynamics may be associated with the diversification of cycads.

## Significance Statement

Telomeric sequences, typically found at chromosome ends, play an essential role in genome integrity. We studied their abundance across land plants and observe large blocks of telomeric repeats at centromere positions, particularly on telocentric chromosomes of cycads. Their comparisons with patterns of chromosome divergence provide evidence for ancient chromosome fission events in cycad ancestries.

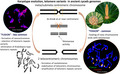

## INTRODUCTION

Species belonging to the Cycadales (cycads) comprise an ancient lineage that diverged from other seed plants at about 300–280 million years ago (MYA) during the Pennsylvanian period (Norstog & Nicholls, 1997). Although the extant cycads phenotypically resemble those found in ancient fossils, genetic data indicate that the group radiated relatively recently, within the last 19 MYA (Condamine et al., 2015; Nagalingum et al., 2011; Renner, 2011). Phylogenetic analysis has shown that cycad species are divided into two families, the Cycadaceae and Zamiaceae (Chaw et al., 2005). Cycadaceae contain just one genus, *Cycas*, with about 118 species (Calonje et al., 2022). All its members display morphological, cytological and cytogenetic similarities indicating low diversity of the group. The more diverse Zamiaceae family comprises nine genera (249 species): *Bowenia* (two), *Ceratozamia* (36), *Dioon* (18), *Encephalartos* (65), *Lepidozamia* (two), *Macrozamia* (41), *Microcycas* (one), *Stangeria* (one), and *Zamia* (83) (Calonje et al., 2022). Like other ancient groups, existing cycad taxa are the result of long evolutionary histories with long periods of reproductive isolation and multiple extinctions (Condamine et al., 2015; Renner, 2011). They have long been considered as a group without polyploidy (Gorelick & Olson, 2011; Levin & Wilson, 1976). Nevertheless, recent whole genome sequencing of *Cycas panzhihuaensis* indicate ancestral gene duplications (Liu et al., 2022), which probably occurred with a whole genome duplication event in the ancestry of all gymnosperms (Li et al., 2015). Thus, there is no evidence for recent (<200 MYRs) polyploidy in cycads.

Cycadaceae include species with highly asymmetrical karyotypes (2*n* = 2*x* = 22) harboring near equal numbers of telocentric (t) and meta‐/submetacentric (m/sm) chromosomes (Kokubugata & Kondo, 1996; Rastogi & Ohri, 2020). In Zamiaceae the basic chromosome number is variable, ranging from *x* = 8 (*Stangeria*, *Ceratozamia*) up to *x* = 14 in some *Zamia* species (Caputo et al., 1996; Moretti, 1990; Rastogi & Ohri, 2020). Several studies have proposed that Robertsonian fusions/rearrangements have contributed to the karyotypic variability in this group (Caputo et al., 1996; Ehrendorfer, 1976; Leitch & Leitch, 2012; Olson & Gorelick, 2011). The chromosomes of cycads have been analyzed by molecular cytogenetics using fluorescent *in situ* hybridization (FISH), with classical ribosomal DNA (rDNA) probes typically showing multiple 35S rDNA loci (Hizume et al., 1992; Tagashira & Kondo, 2001) and sequence analysis revealing high levels of rDNA pseudogenization (Wang et al., 2016). As in other gymnosperms, genome sizes are relatively large, ranging from 12.5 to 31.7 (21.3 average) Mb per haploid genome (Zonneveld, 2012; Zonneveld & Lindstrom, 2016).

Eukaryotic linear chromosome ends are characterized by telomeres, which are typically composed of long tracts of simple tandem repeats (Peska & Garcia, 2020; Ruiz‐Herrera et al., 2008). Telomeres are maintained by telomerase, a reverse transcriptase‐based end maintenance system (Wu et al., 2017). Telomeres help to prevent the loss of essential genetic information from the ends of chromosomes, resulting from the incomplete replication of chromosomal DNA, and help to avoid detrimental chromosomal end‐to‐end fusions (van Steensel et al., 1998). Thus, telomeres maintain the integrity and stability of chromosomes. The telomere repeat motif 5′‐(TTTAGGG)n‐3′ (henceforth called “TTTAGGG”), discovered in *Arabidopsis thaliana* (Shakirov & Shippen, 2004), has been considered the consensus telomere motif for most land plants. However, some plant lineages have been shown to use other variant motifs (i.e., TTAGGG, TTTTTAGGG, CTCGGTTATGGG) to protect their chromosome ends (Adams et al., 2000; Fajkus et al., 2016; Peska et al., 2015; Peska & Garcia, 2020; Shibata & Hizume, 2011; Weiss‐Schneeweiss et al., 2004). In angiosperms the total amount of telomeric repeats determined by gel electrophoresis varies by orders of magnitude, ranging from several hundred bp up to tens of kb per species (Fajkus et al. 1995; Shakirov & Shippen, 2004).

Telomeric sequences have occasionally been reported to occur at non‐telomeric positions, although such loci, collectively called interstitial telomeric repeats (ITR), are more common in animals than in plants (Ruiz‐Herrera et al., 2008). Nevertheless, several publications have demonstrated the presence of ITS in various phylogenetically diverse plant species (Fuchs et al., 1995; Majerova et al., 2014; Mlinarec et al., 2019; Rosato et al., 2018; Souza et al., 2016). A systematic review revealed ITRs of sufficient size that are resolvable using cytogenetics are found in nearly all major lineages of seed plants (Maravilla et al., 2021). The number of these ITR sites varied from a single locus up to more than 70 sites in some karyotypes. It has been proposed that such sites may represent remnants of chromosome fusions, recombination, and chromosome breakages (Fonseca & Pedrosa‐Harand, 2013; Grabowska‐Joachimiak et al., 2015; Guerra, 2008; Lysak et al., 2016; Ruiz‐Herrera et al., 2008; Schubert & Rieger, 1990; Slijepcevic, 1998). However, a meta‐analysis of karyotypes showed no apparent association between the presence of ITR signals and the number of chromosomes (Maravilla et al., 2021). Moreover, highly dynamic ITR sites found outside pericentromeric positions in *Anacyclus* exhibit large inter‐ and even intra‐species variability (Rosato et al., 2018). Telomeric repeats may also mutate and eventually evolve into satellite DNA at non‐telomeric positions (Emadzade et al., 2014; Gao et al., 2022; Mlinarec et al., 2009). These observations argue against Robertsonian fusions/rearrangements being a sole mechanism of ITR origins and alternative hypotheses were proposed, i.e. (i) the repair mechanisms of DNA double‐strand breaks (Ruiz‐Herrera et al., 2008), and (ii) integration of telomeric extrachromosomal covalent circles (eccDNA) (He et al., 2013; Rosato et al., 2018). Based on the above evidence, ITR may have arisen through multiple mechanisms in land plants.

In this work, we undertake a detailed genomic analysis of the organization of telomeric repeats, their abundance and homogeneity in cycads and compare these data with a wide range of land plant species. We exploit available genomic resources as well as generating *de novo* sequence data from 29 gymnosperm and one angiosperm species. Variant‐specific FISH is used to determine the physical position of telomeric repeat variants in cycad chromosomes, while the positions of centromeres in cycad karyotypes are unambiguously identified by immunohistochemical staining using an antibody against the phosphorylated serine 10 histone H3 protein (H3S10P). By analyzing telomeric repeat dynamics in the highly asymmetrical cycad genomes, evidence is provided to show that frequent Robertsonian rearrangements have indeed impacted the evolution of their chromosomes.

## RESULTS

### Identification and quantification of telomeric repeats in high‐throughput reads

**Figure 1:**
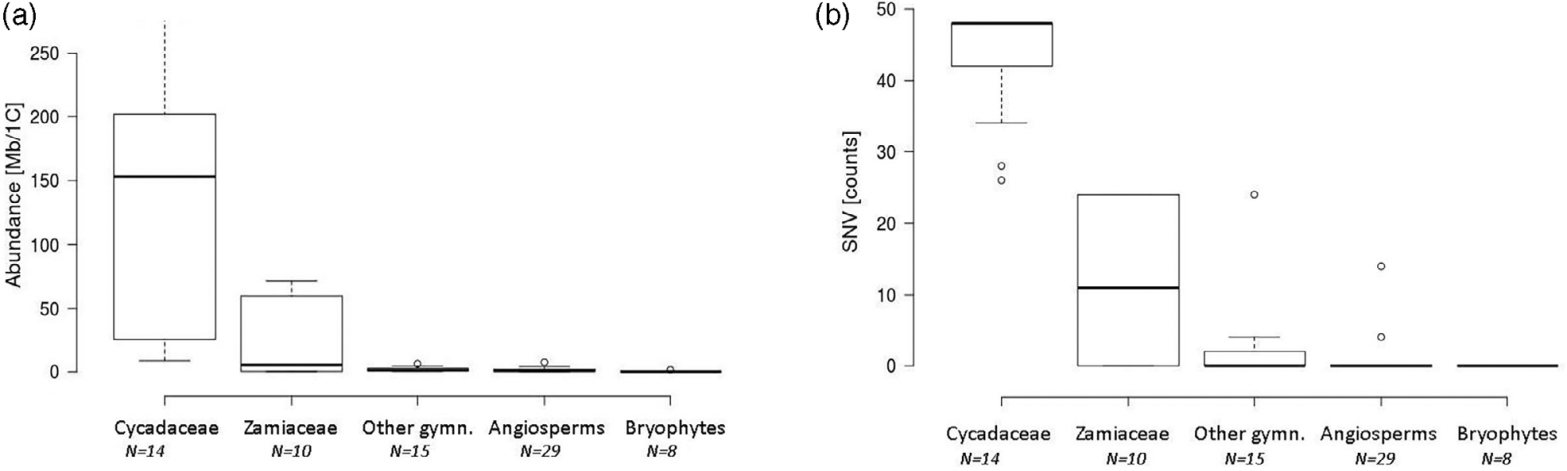
The abundance and diversity of telomeric repeats in plant genomes. (a) Genomic abundance of telomeric repeats calculated in Mb/1C. Boxes represent the second and third quartiles; horizontal line is the median value. (b) Intragenomic heterogeneity of telomeric repeats expressed as the number of single nucleotide variants (SNVs) in mapped reads occurring at ≥20% frequency. The reference was a 14‐mer of the *Arabidopsis*‐type telomeric repeat. gymn., gymnosperms.

From sequence read archives and newly sequenced genomes, we analyzed telomeric repeat abundance and diversity in 29 cycads (24 newly sequenced), 15 other gymnosperms (five newly sequenced), 29 angiosperms (one newly sequenced), and eight bryophytes (Tables S1 and S2). The abundance of the *Arabidopsis*‐type telomeric (TTTAGGG)n repeat motif was estimated from the number of mapped reads relative to total reads and expressed in Mb/1C (Figure 1a, Figures S1) and genome proportion (GP) (Figures S1). There was a correlation between absolute amounts of repeats and their GP (Pearson's, *r* = 0.930, Table 1) and no correlation with genome size (Pearson's, *r* = 0.196; *P* > 0.05, Table 1). Large interspecific differences were observed in telomeric repeat abundance. Among gymnosperms, the highest abundance of telomeric repeats was found in *Cycas changjiangensis* (278 Mb/1C), with the lowest in *Dioon spinolosum* (0.13 Mb/1C). Among angiosperms, the highest abundance was observed in *Anacyclus pyrethrum* (Asteraceae, 8.7 Mb/1C) while the lowest was in *Genlisea nigrocaulis* (Lentibulariaceae) and *Cardamine amara* (Brassicaceae), both with 0.03 Mb/1C. The species with the largest genomes analyzed (>35 Gb/1C), i.e., *Fritillaria imperialis* and *Lilium tsingtauense* contained a relatively low abundance of telomeric repeats (2.47 and 2.16 Mb/1C, respectively; GP = 0.0058% for both species). In bryophytes, the highest abundance of telomeric repeats was found in the liverwort *Pallavicinia lyelli* (1.47 Mb/1C), while the lowest was in the moss *Polytrichum formosum*, hornwort *Anthoceros punctuates*, and liverwort *Marchantia polymorpha* (all 0.02 Mb/1C). It is evident that many of the cycads analyzed showed a considerably higher abundance of telomeric repeats compared with the other land plant groups investigated (Figure 1a).

**Table 1:** Statistical evaluation of the relationships between telomeric repeat abundance, diversity, genome size, and chromosome morphology

Variable 1	Variable 2	Number of values	Pearson's *r* ^a^	Spearman's rho^a^
Telomeric repeats (Mb/1C)	Telomeric repeats (GP)	77^b^	**0.93025**	**0.80056**
Telomeric repeats (Mb/1C)	Telomeric repeats diversity (SNV)	77^b^	**0.77716**	**0.79792**
Telomeric repeats (Mb/1C)	Genome size (Mb/1C)	77^b^	0.19639	0.54212
Number of chrom./2C	Number of T^d^ chrom./2C	96^c^	**0.94870**	**0.82183**
Number of chrom./2C	Genome size (Mb/1C)	48^c^	−0.32977	−0.18454
Number of T^d^ chrom./2C	Genome size (Mb/1C)	48^c^	−0.39748	−0.26831

^a^ Strong relationship is indicated by correlation coefficients ≥0.7 (in bold).

^b^ Data are taken from Table S2.

^c^ Data are taken from Table S3.

^d^ Number of telocentric chromosomes in karyotypes.

### *In silico* identification of telomeric repeat variants

**Figure 2:**
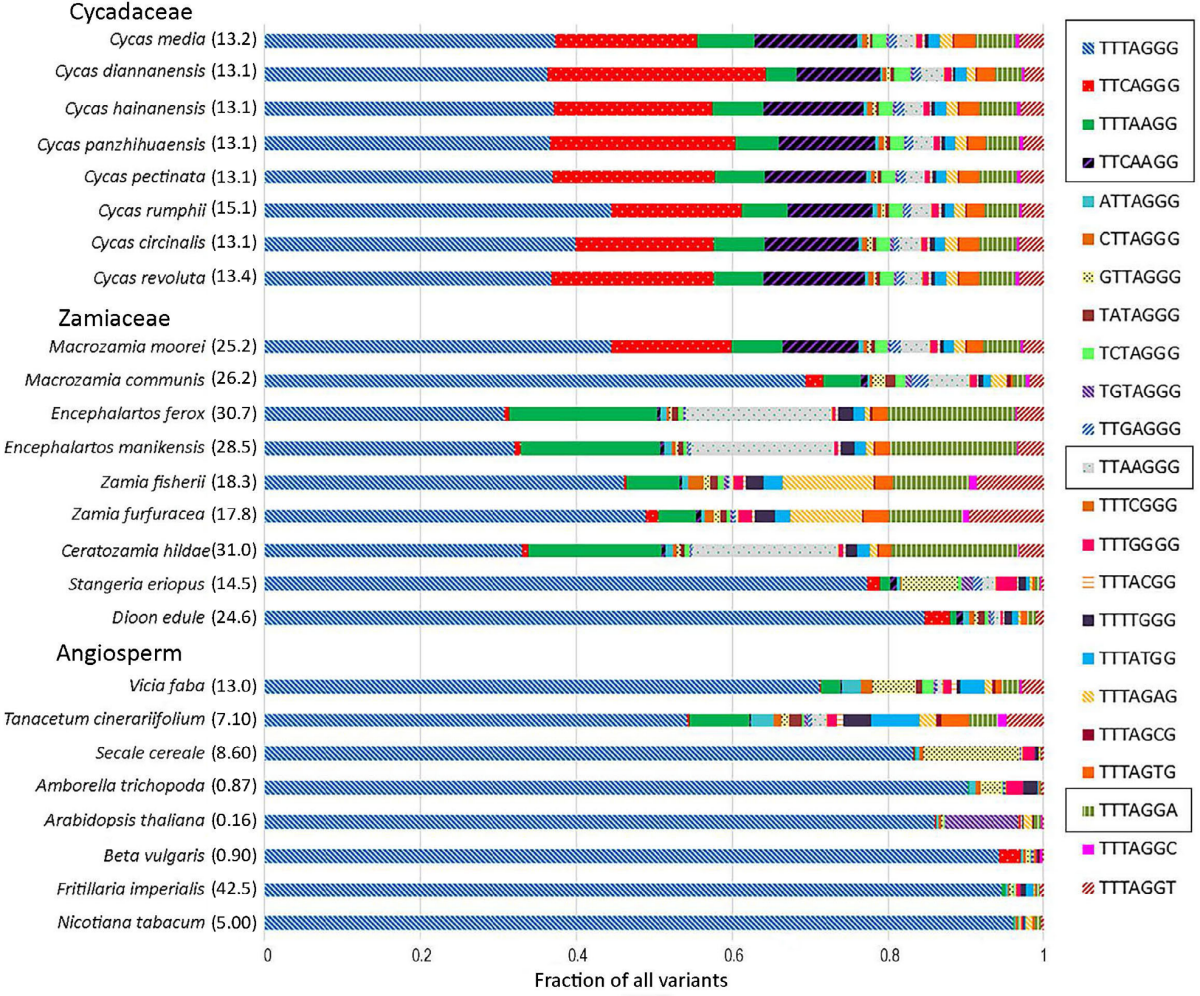
Genomic representation of telomeric repeat variants. Stacked graphs are showing the frequencies of all 22 possible variants that differ from the canonical TTTAGGG motif by a single substitution, and the TTCAAGG motif, which differs by two substitutions. Typically, the data were obtained from the analysis of several thousand reads. Note the uniform profiles in Cycadaceae and a more variable spectra in Zamiaceae. The most abundant variants are enclosed within boxes in the key on the right‐hand side. The values in parenthesis show the genome size (Gb/1C). Two main variants (TTTAGGG and TTCAGGG) were analyzed by Southern blot hybridization (Figure S2) and FISH (Figures 3 and 4).

The sequence diversity of telomeric repeats was determined by single nucleotide variant (SNV) analysis of mapped reads (Figure 1b and Table S2) revealing a large number of SNVs in mapped telomeric reads from cycads. There was a significant correlation between telomeric repeat abundance and repeat diversity (Pearson's, *r* = 0.777; *P* < 0.001, Table 1). To determine the variant composition of arrays, we searched the genomes for telomeric motif variants differing from the *Arabidopsis* type TTTAGGG motif by a single substitution. Eight representative species each from Cycadaceae, Zamiaceae, and angiosperms (from different families) were selected. Species expected to contain non‐*Arabidopsis*‐type telomeric repeats were not considered (Peska et al., 2015). Through this approach, several abundant (occurring at ≥10% frequency) variants of the telomeric heptanucleotide motif were identified (Figure 2). In all species the *Arabidopsis*‐type TTTAGGG was the most abundant variant, accounting for 30%–98% of all variants. However, in *Cycas*, three additional variants (TTCAGGG, TTTAAGG, and TTCAAGG) were found in high proportions. Of these, the TTCAGGG variant was the second most abundant motif, accounting for 22%–31% of all variants in the genomes analyzed. Among Zamiaceae, for *Dioon edule* and *Stangeria eriopus* the canonical TTTAGGG motif was the most common, comprising over 75% of all variants, while both *Encephalartos* species contained other abundant variants, with the TTAAGGG motif being the most abundant (18%). In angiosperms the *Arabidopsis*‐type TTTAGGG repeat predominated (i.e., >80% of the variants) in six of the eight species analyzed, with the exception being in *Tanacetum cinerariifolium* (Asteraceae), which contained a greater proportion of other variants.

### Southern blot hybridization analysis of telomeric variants

To confirm the presence of telomeric variants in the cycad genomes, we carried out Southern blot hybridization analysis of terminal telomeric fragments. Genomic DNAs from gymnosperm and angiosperm species were digested with *Hae*III, *Taq*I, and *Tru*1I restriction enzymes, which cut tetranucleotide motifs outside the TTTAGGG sequence (Figure S2). In the angiosperm *Nicotiana tabacum* (tobacco) and the gymnosperms *D. edule*, *Dioon spinulosum*, and *Welwitschia mirabilis*, the probe only hybridized to the high molecular weight DNA fragments indicating a lack of digestion of telomeric arrays with any of the enzymes used. In contrast, *Tru*1I cutting at the TTAA motif efficiently digested telomeric repeats in both *Cycas revoluta* and *Cycas circinalis*. Thus, the presence of the TTTAAGG variant was confirmed in the two *Cycas* genomes. As there was no undigested relic DNA in *C. revoluta*, this indicates that the TTTAGGG motif was largely interspersed within other telomeric variants. In contrast, about half of the *C. circinalis* fragments remained undigested with *Tru*1I, indicating higher homogeneity of telomeric repeats in this species. Partial digestion of *Cycas* DNA was also obtained with *Taq*I (TCGA motif) while *Hae*III (GGCC) did not digest telomeric arrays.

To compare the methylation status of the divergent telomeric repeats in *C. revoluta* and *N. tabacum* (tobacco), which harbor long homogeneous arrays of *Arabidopsis*‐type (TTTAGGG) repeats, genomic DNA of each species was digested with the McrBC enzyme, which will only cuts sequences containing methylated cytosine residues. The DNAs were then run on agarose gels and hybridized with probes against the *Arabidopsis*‐type motifs (Figure S3a) and its TTCAGGG variant (Figure S3b). From the profiles of both probes, it is evident that the McrBC enzyme efficiently digested the *C. revoluta* DNA into a smear of fragments of about 0.2–2 kb in size leaving no significant signal in the high molecular weight fraction. Such results indicate high levels of cytosine methylation in telomeric arrays. In contrast to *C. revoluta*, tobacco telomeric TTTAGGG repeats showed little or no digestion with McrBC, indicating low levels or no cytosine methylation. The TTCAGGG probe did not hybridize with tobacco DNA at all, confirming the absence of this telomeric variant in the species.

### Identification of cycad centromeres by immunostaining of chromatin

**Figure 3:**
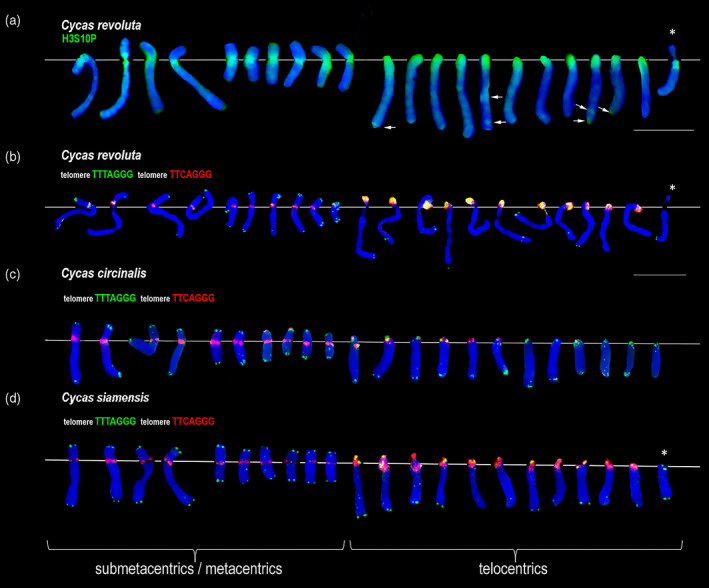
FISH and immunostaining analysis of *Cycas revoluta*, *Cycas circinalis* and *Cycas siamensis* metaphase chromosomes. (a) Chromosomes immunostained with an antibody against phophorylated histone H3S10P (in green). Note, specific staining of centromeres in metacentric and telocentric chromosomes. Arrows indicate faint immunostaining of the antibody at interstitial sites. The last chromosome in the row may represent a Y chromosome. (b) *C. revoluta*, (c) *C. circinalis*, and (d) *C. siamensis* chromosomes hybridized with the telomeric TTTAGGG (green) and TTCAGGG variant (red) probes. Colocalized signals of both probes appear yellow–orange. Chromosomes were excised from metaphase pictures (Figure S3) and aligned at the centromeres (thin line). Bar = 10 μm.

**Figure 4:**
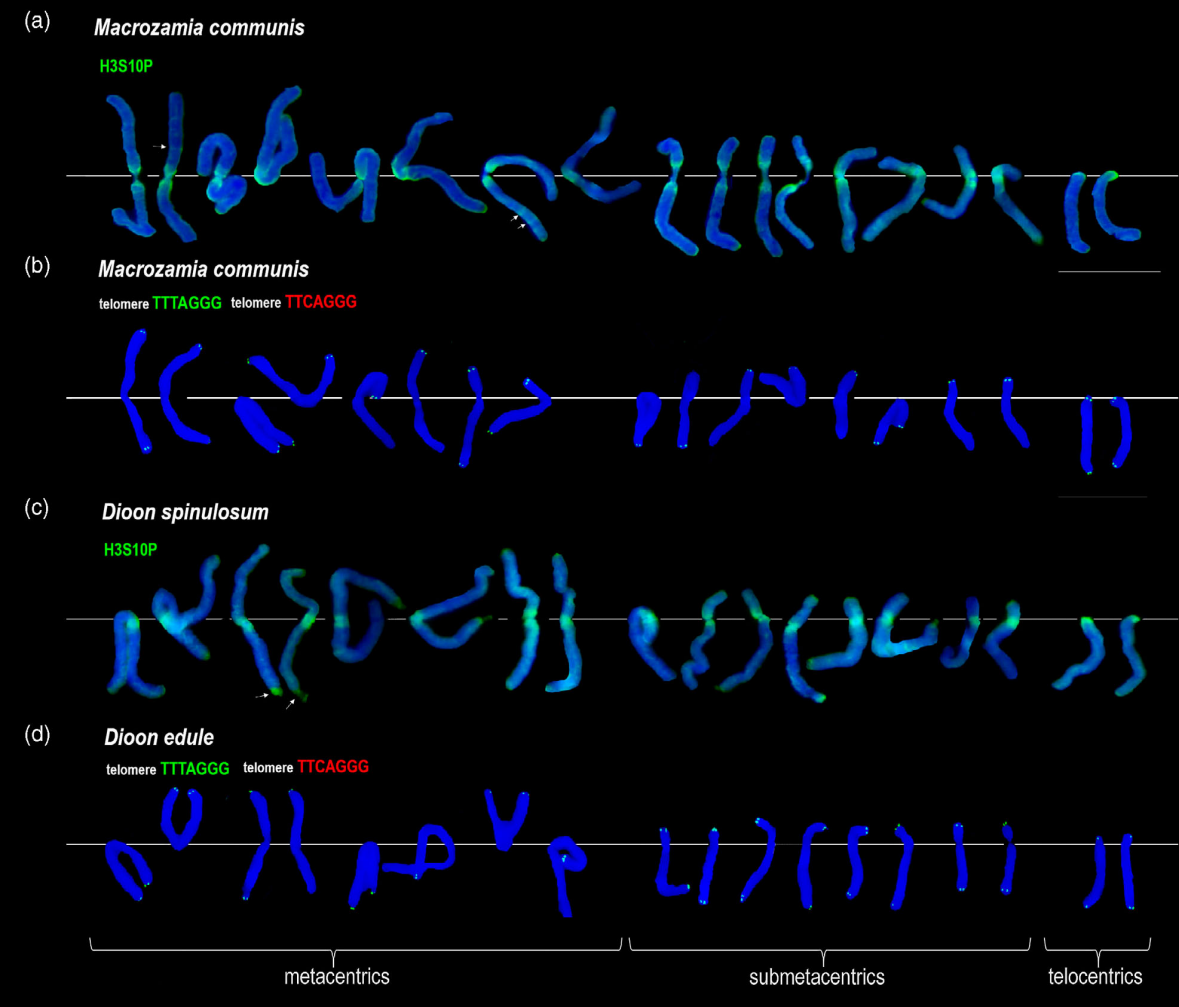
FISH and immunostaining analysis of metaphase chromosomes of species from Zamiaceae. (a,c) Chromosomes immunostained with an antibody against phosphorylated histone H3S10P (in green). Arrows indicate faint immunostaining of the antibody at interstitial sites. (b,d) FISH to metaphase chromosomes with the telomeric TTTAGGG (in green) and TTCAGGG (in red) variant probes. (a,b) *Macrozamia communis*, (c) *Dioon spinolusum*, and (d) *Dioon edule*. Only the TTTAGGG and not TTCAGGG probe hybridized. Bar = 10 μm.

Chromosome numbers differ substantially in Cycadales (Table S3), ranging from 16 to 28 per diploid set. To identify the pericentromeric chromatin in cycad chromosomes we used immunostaining with an antibody against a phosphorylated serine residue of histone H3 (H3S10P). In *C. revoluta* (2*n* = 22), the antibody stained pericentromeric regions on 10 meta‐ and submetacentric and 11 telocentric and one acrocentric (possible Y candidate, asterisks) chromosomes (Figure 3a). The rest of the chromosomes were not heavily labeled, except for faint interstitial smears of signal visible on some chromosome arms. In both Zamiaceae species (*Macrozamia communis* and *D. spinulosum*; each with 2*n* = 18) the anti‐H3S10P antibody stained pericentromeric regions of 16 submetacentric and two telocentric chromosomes (Figure 4a,c). Thus, members of Cycadaceae and Zamiaceae families differ substantially in both chromosome number and types.

Chromosomes of *C. revoluta*, *M. communis*, *D. edule*, and *Zamia furfuracea* were further immunostained with an antibody to 5‐methylcytosine (5‐mC; Figure S4). In *C. revoluta*, the 5‐mC signals appeared to be clustered in pericentromeric regions of most telocentric chromosomes. In contrast, in *M. communis*, *D. edule*, and *Z. furfuracea*, the immunolocalization of 5‐mC was more diffuse, with no clear distinction between pericentromeres and the rest of the chromosomes.

### FISH analysis of telomeric variants

To determine the position of two different telomeric repeat variants on *Cycas* chromosomes we carried out FISH using labeled oligonucleotide probes derived from the *Arabidopsis*‐type TTTAGGG unit and its TTCAGGG variant (green and red signals, respectively, in Figure 3b,c,d). In *C. revoluta*, *C. circinalis*, and *Cycas siamensis* all telocentric chromosomes showed overlapping signals of both probes (yellow–orange signals) in proximal/distal regions. In contrast, there was little overlap between both probes on metacentric chromosomes. In these chromosomes, pericentromeric regions mostly hybridized with the TTCAGGG probe while telomeres stained with the TTTAGGG probe (see Figure S5 for the signals displayed separately). A putative *C. revoluta* and *C. siamensis* Y chromosome (Figure 3b,d, asterisks) showed hybridization with the TTTAGGG probe at telomeric positions consistent with the previous study (Hizume et al., 1998). A further three cycad species, *Cycas deboanensis*, *Cycas multipinnata*, and *Cycas multifrondis* were also hybridized with the TTTAGGG probe. In all species, the probe hybridized strongly to proximal regions of telocentric chromosomes (Figures S6 and S7). However, there were only faint signals to telomeric regions in *C. deboanensis*, *C. multipinnata*, and *C. multifrondis*, which might be due to a low abundance of telomeric repeats and/or differences in chromosome size. In *C. multipinnata* interphase nuclei, the proximal telomeric sites tend to group at one pole of the nucleus (Figure S6b,c). *Macrozamia communis* and *D. edule* both showed hybridization with TTTAGGG, but not TTCAGGG probes (Figure 4b,d and Figure S5), with the TTTAGGG probe hybridizing only to the chromosome ends. Similarly, *Z. furfuracea* showed standard localization of TTTAGGG sites on chromosomal ends (Figure S7c). Table 2 summarizes information about the karyotypes and hybridization patterns.

**Table 2:** Summary of fluorescent *in situ* hybridization analysis

Species	Karyotype		Number and position of telomeric repeat variants			
	2*n*	Type	TTTAGGG		TTCAGGG	
			Proximal	Distal	Proximal	Distal
*Cycas circinalis*	22	2m	0	4	2	0
		4sm	0	8	4	2
		4st	2 (w)	8	4	0
		12t	12	12	7	0
*Cycas debaonensis*	22	12t	10–12 (s)	U	NA	NA
		10 others	8‐10 (w)	U	NA	NA
*Cycas multifrondis*	22	12t	10 (s)	U	NA	NA
		10 others	6 (w)	U	NA	NA
*Cycas multipinnata*	22	12t	10–12 (s)	U	NA	NA
		10 others	8 (w)	U	NA	NA
*Cycas revoluta*	22	4m	4	8	4	0
		2sm	2	4	2 (s)	0
		4st	2–4 (2w)	8	4	0
		12t	11 (s)	8–10	11 (8s)	0
*Cycas siamensis*	22	2m	2 (w)	4	2	0
		4sm	4 (w)	8	4	0
		4st	4 (w)	8	4	0
		12t	11	11	11 (s)	0
*Dioon spinulosum*	18	8m	0	14	0	0
		8st	0	16	0	0
		2t	0	4	0	0
*Macrozamia communis*	18	8m	0	16	0	0
		8sm	0	9–16	0	0
		2t	0	4	0	0
*Zamia furfuracea*	18	4t	0	8	NA	NA
		14 others	0	28	NA	NA

Terminology follows the nomenclature of (Caputo et al., 1996; Kokubugata & Kondo, 1996; Rastogi & Ohri, 2020).

m, metacentric; NA, not analyzed; s, strong signals; sm, submetacentric; st, subtelocentric; t, telocentric chromosomes; U, undetectable signals; w, weak signals.

### Evolution of chromosome numbers and genome sizes across cycads

**Figure 5:**
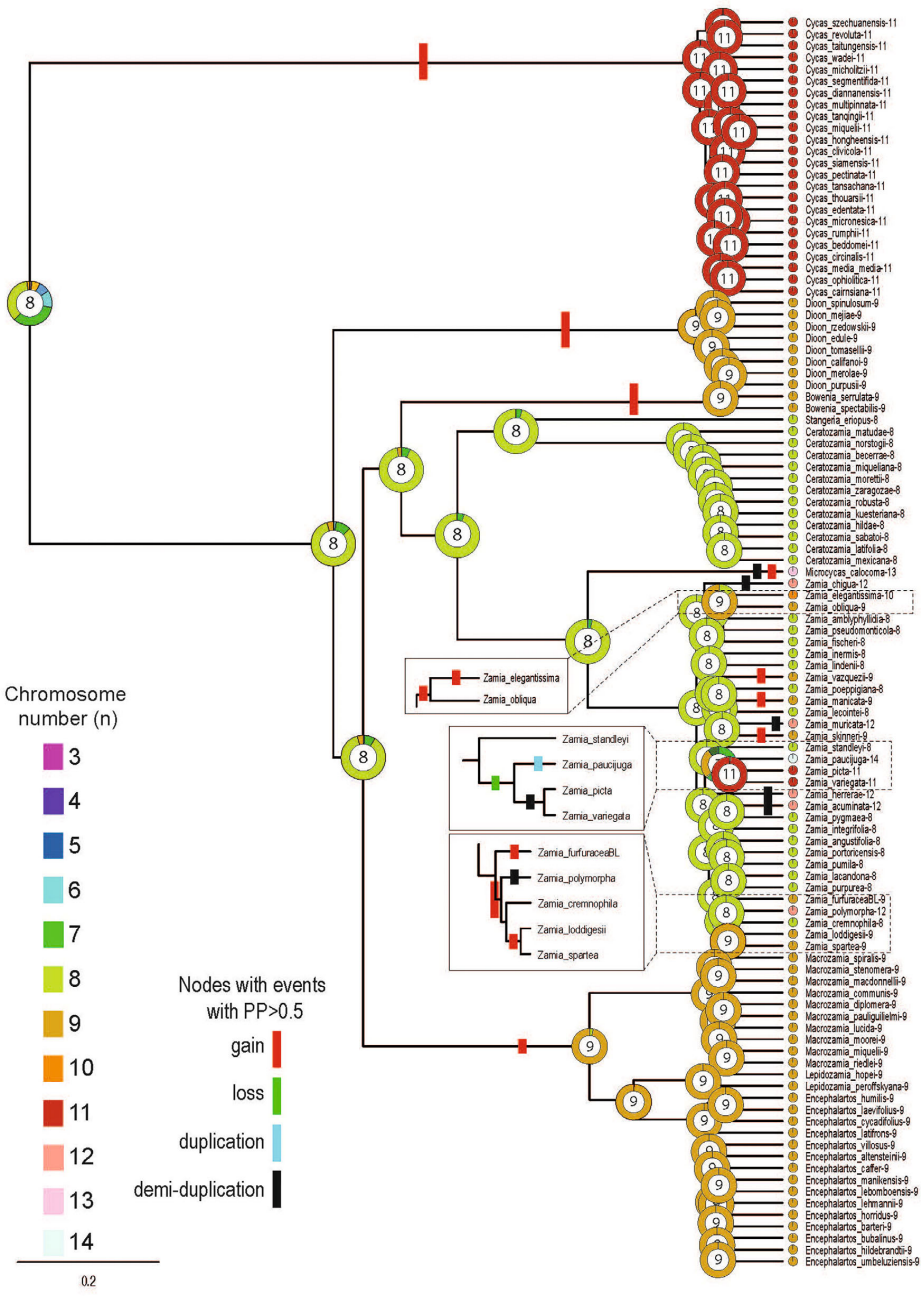
Phylogenetic tree showing the evolution of cycad species combined with ancestral chromosome number reconstruction. Number of chromosomes in the species analyzed ranged from *n* = 8 to *n* = 14. Each outer circle shows the posterior probability (PP) of different chromosome numbers (*n*) (see color key), with the chromosome number with the highest posterior probability shown in the center of each circle. Note, the lack of diversification in the chromosome number within Cycadaceae in contrast to the diversity observed within *Zamia* (Zamiaceae).

Ancestral reconstruction of chromosome numbers in ChromEvol supported the three‐parameter model (M2), with constant rate and demi‐duplications, being the best‐fit model (Akaike Information Criterion = 188.2) to our data. The ancestral haploid chromosome number for all cycads reconstructs close to *n* = 8, although with a relatively low posterior probability (PP = 0.36, Figure 5). We were unable to reconstruct an ancestral chromosome number for cycads as *n* = 11 using the methods published in Gorelick et al. (2014).

**Figure 6:**
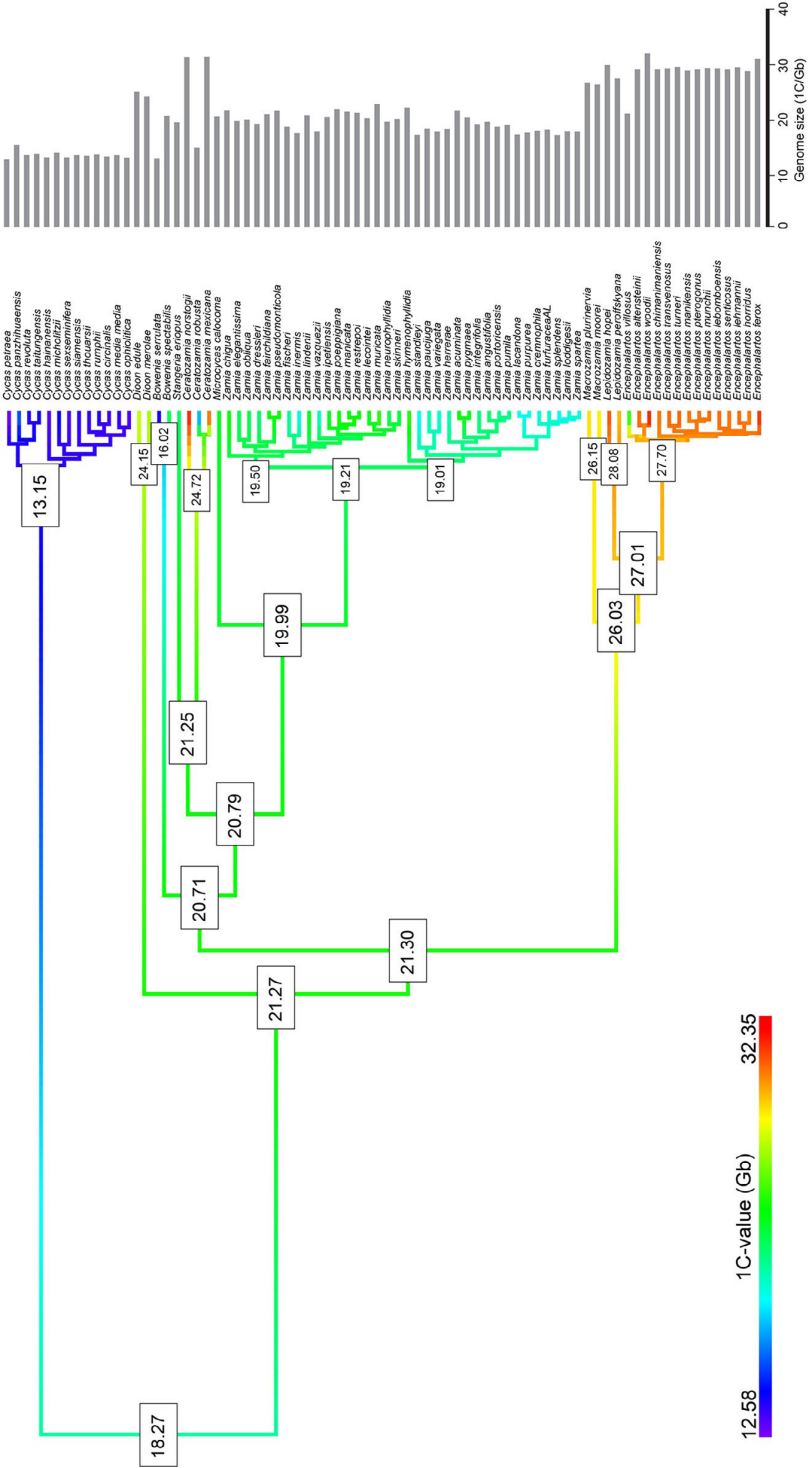
Ancestral genome size reconstruction (Gb/1C) in cycads. Branch colors represent ancestral genome sizes estimates. Values obtained for the most recent common ancestors of relevant nodes are depicted in the phylogenetic tree.

The genome size for the most recent common ancestor (MRCA) of Cycadales was reconstructed at about 18 Gb/1C (Figure 6). Despite extant representatives of *Cycas* having the smallest genome sizes across Cycadales (Table S2), an increase in the chromosome number up to *n* = 11 along the branch leading to Cycadaceae was inferred (i.e., *Cycas*, all of which are 2*n* = 22). The MRCA of all remaining ancestors also reconstructed as *n* = 8, diverging from this to *n* = 9 in the clade comprising *Macrozamia*, *Lepidozamia*, and *Encephalartos* and independently in the separate clades leading to *Bowenia* and *Dioon* (Figure 5). Unlike *Cycas*, the divergence of the remaining species is associated with genome expansion, particularly in the clade comprising *Macrozamia*, *Lepidozamia*, and *Encephalartos* (Figure 6), whose MRCA is reconstructed with a genome size of 26.03 Gbp/1C.

An ancestral chromosome number of *n* = 8 (PP = 0.94) was reconstructed on the branch leading to *Stangeria* and *Ceratozamia*, and its sister clade comprising species in *Zamia* and *Microcycas* (Figure 5). The divergence of *Microcycas* and *Zamia* was however associated with complex chromosome number changes, giving rise to *n* = 13 (*Microcycas*) and a range of chromosome numbers that have been reported in *Zamia* (from *n* = 8–14 [excluding *n* = 10]) (Caputo et al., 1996; Kokubugata et al., 2000; Rastogi & Ohri, 2020; Tagashira & Kondo, 2001). Independent fissions already occurred early in the divergence of *Zamia* giving rise to *n* = 9 species (Figure 5). Thereafter, depending on species lineages further fusions and fissions are reconstructed to give the full range of chromosome numbers reported in *Zamia* (Marchant, 1968; Moretti, 1990; Rastogi & Ohri, 2020).

**Figure 7:**
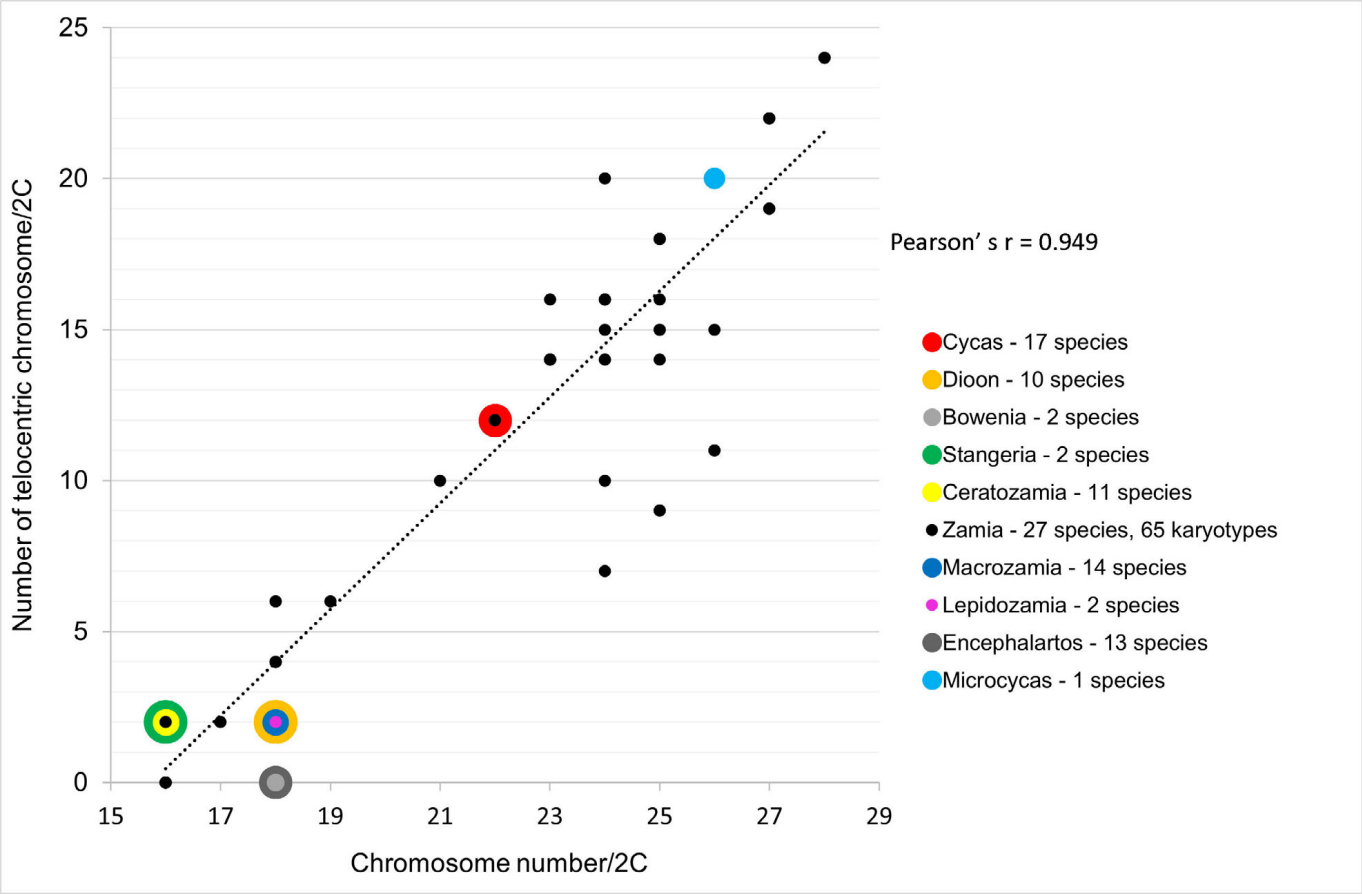
Relationship between chromosome number and number of telocentric chromosomes in cycads. Data were compiled from Table S3. Number of species in overlapping data points are reflected by the size of marks.

Despite the complexity in *Zamia*, the overall pattern of genome divergence in cycads is one of increased chromosome number (Figure 5). There is a relationship (Pearson's *r* = 0.949) between chromosome number and number of telocentric chromosomes in cycad karyotypes (Figure 7), indicative of centric chromosome fissions being involved in chromosome number increases. The relationship between genome size and chromosome number is ambiguous in cycads as there might be genome upsizing (most members of the Zamiaceae family) and genome downsizing (all members of the Cycadaceae family).

## DISCUSSION

### Variable abundance of telomeric repeats in plant genomes

Telomeric repeats have been extensively studied by cytogenetic and molecular biology methods (Maravilla et al., 2021; Peska & Garcia, 2020), but less commonly using genomic approaches. Here we analyzed their abundance and homogeneity in a wide range of land plants using high‐throughput sequencing and molecular cytogenetic methods. We found that their abundance and sequence heterogeneity was similar and relatively low in all angiosperms and bryophytes analyzed compared with gymnosperms, which exhibited large variations in both characters (Figure 1, Table S2). Indeed, there was an extraordinarily high abundance and diversity of telomeric variants in *Cycas*, in which Mb‐sized telomeric blocks are found at both centromeric and telomeric domains. In general, the telomeric repeat abundance was positively correlated with telomeric array heterogeneity and their occurrence at non‐telomeric positions. It is well‐established that non‐coding repetitive DNA sequences constitute a major fraction of many plant genomes and largely account for interspecies differences in genome size (Bennett & Leitch, 2011; Bennetzen & Kellogg, 1997; Novak et al., 2020). However, we found no correlation between the abundance of telomeric repeats and genome size, a trend that it is otherwise typical for other abundant repeats (Novak et al., 2020). Similarly, no correlation was observed between the number of ITR and chromosome numbers (Maravilla et al., 2021). Potentially, telomeric repeat abundance reflects chromosome dynamics in telomeric regions, as well as the history of breakages, inversions, fusion, and fissions in a species' ancestry (Maravilla et al., 2021; Ruiz‐Herrera et al., 2008; Slijepcevic, 1998), which is the rate at which such chromosome rearrangements may occur independently of genome size and chromosome numbers.

Traditionally, the lengths of telomeric repeats have been determined based on size separation of restriction fragments by electrophoresis or *in silico* approaches, although the estimates differ considerably. For example, the average size of *N. tabacum* (tobacco) telomeric fragments determined by pulsed field electrophoresis was approximately 90 kb/chromosome, corresponding to a total of approximately 2.2 Mb of telomeric DNA per haploid (1*n* = 24) genome (Fajkus et al., 1995). In contrast, the calculated genome abundance of tobacco telomeric sequences from genomic data presented was estimated at about 4.0 Mb (see Table S2). Similarly, the total amount of *A. thaliana* telomeric repeats based on the average size of telomeric fragments of *A. thaliana* (Shakirov & Shippen, 2004) was estimated to be four‐fold lower than the estimated value from *in silico* analyses of genomic sequences (this work, Table S2). The differences likely arise from variable numbers of telomeric repeats and their degenerative variants scattered across the genome, outside of the telomeres themselves. In fact, blurred signals of the telomeric probe hybridization observed in some Southern hybridization experiments (Fojtova et al., 2015) support this suggestion.

### Origin of telomeric repeat variants in cycad genomes

The diversity of cycad telomeric repeats was remarkable (Figure 2). In *Cycas*, four major variants were amplified and organized in Mb‐sized blocks (i.e., TTTAGGG, TTTAAGG, TTCAGGG, and TTCAAGG). While TTCAGGG and TTCAAGG were particularly abundant in *Cycas*, the TTTAAGG variant was abundant in some of the Zamiaceae species analyzed. Expansion of the TTCAGGG variant may explain the GC‐rich bands that have been observed in these regions after chromomycin A staining (Kokubugata & Kondo, 1996). No significant amounts of TTAGGG repeats, typical of vertebrates, were found in any of the gymnosperm genomes analyzed, confirming their relatively low abundance or absence, as previously reported (Shibata & Hizume, 2011). With the possible exception of *T. cinerariifolium*, we failed to detect significant telomeric repeats heterogeneity in species with cytogenetically confirmed interstitial and proximal located telomeric repeats (Aronen & Ryynanen, 2012; Fuchs et al., 1995; Mlinarec et al., 2019; Rosato et al., 2018).

The diversity of telomeric repeats we observe in cycads could potentially be generated by telomerase RNA activities, which may differ between gymnosperms and angiosperms (Song et al., 2019). However, several lines of evidence indicate that the amplified telomeric variants identified in our paper are unlikely to be the products of distinct telomerase activities, i.e., (i) in Cycadaceae, the TTCAGGG variant appears to be localized in pericentromeric regions, while the canonical TTTAGGG variant occurs at both centromeric and telomeric positions (Figure 3), and (ii) the telomeric arrays are highly heterogeneous in *C. revoluta* (Figure S8), and different variants tend to intermingle (Data S1) consistent with a previous analysis based on one clone (Hizume et al., 1998). It is therefore most likely that the telomeric repeat heterogeneity observed in cycads arises from molecular processes acting on canonical telomeric repeats after their translocation to non‐telomeric positions. For example, it is known that centromeric/pericentromeric repeats are particularly prone to recombination‐based gene conversion events (Talbert & Henikoff, 2010) and it is not uncommon to observe the same type of repeat on different chromosomes (Heslop‐Harrison & Schwarzacher, 2011). Indeed, the TTCAGGG variant seems to be present on all chromosomes of *C. revoluta* and *C. circinalis*, and FISH experiment indicates distinct spatial orientation of telocentric chromosomes, which seem to cluster at interphase (Figure S6b,c). Perhaps, clustering of centromeres may lead to physical interaction that stimulates gene conversion and homogenization of centromeric repeats across the genome (Talbert & Henikoff, 2010). However, individual telomeric variants might have arisen through diverse genetic processes. For example, gene conversion and associated DNA repair could also lead to the accumulation of GC sequences (Marais, 2003), and might have changed TTTAGGG into the TTCAGGG motif by a T>C substitution in the third nucleotide position. Variants could also arise by cytosine deamination (C>T substitution) of the third cytosine on the opposite DNA strand, which could generate the TTTAAGG variant. The variant is frequently found in gymnosperm and even in two angiosperm genomes analyzed (*Tanacetum* and *Vicia*; Figure 2, green).

### Epigenetic modification of telomeric repeats

Telomeric repeats are known to exhibit numerous unique chromatin features, including short nucleosome repeat lengths and distinct epigenetic modifications (Fojtova & Fajkus, 2020; Vaquero‐Sedas & Vega‐Palas, 2019). At the cytogenetic level, our data show that the pericentromeric blocks of telomeric repeats clearly overlap with the 5‐mC signals in *C. revoluta* (Figure S4a). In contrast, distal positions composed mostly of canonical TTTAGGG sequences are not markedly stained with the anti‐5‐mC antibody. Molecular experiments indicate that the telomeric variants are methylated in this genome (Figure S3). It is likely that the second most common telomeric variant TTCAGGG is preferentially methylated, as it contains a symmetrical CAG motif known to be the target of plant chromomethylases (Meyer, 2011). However, it should be mentioned that strong pericentromeric 5‐mC signals cannot be solely attributed to telomeric variants as these regions are extremely large (below) and may contain rDNA (Wang et al., 2016) and other repeats. Moreover, potentially discontinuous 5‐mC signals were detected in *D. edule*, *M. communis*, and *Z. furfuracea* (Figure S4b,c,d), indicative of regions with variable levels of cytosine methylation. Potentially, high‐resolution mapping of 5‐mC by bisulfite sequencing and/or third‐generation sequencing technologies may help to resolve large‐scale organization of these domains.

The histone H3 protein phosphorylation at the 10th serine residue (H3S10P) specifically marks pericentromeric regions of mitotic chromosomes (Houben et al., 2007). Correspondingly, the H3S10P signals were strongest in the pericentromeric regions in all species. Faint fluorescence signals were also observed at non‐centromeric positions, particularly in *C. revoluta* (Figure 3a), but also on *M. communis* and *D. spinulosum* chromosomes (Figure 4a,c). These sites may represent remnants of former centromeres, neocentromeres, localization of rDNA loci, or technical artifacts of immunostaining. Estimates indicate that the H3S10P signals span, in average, about one‐tenth of the chromosome size in *C. revolute*, which roughly corresponds to more than 100 Mb. Thus, pericentromeric regions are unusually large in this species, particularly those in telocentric chromosomes.

### Chromosome evolution in cycads

The extant cycad genera are thought to have radiated about 250 MYA (based on divergence age estimates for the split between lineages leading to extant Zamiaceae and Cycadaceae (Condamine et al., 2015) and they show significant variation in their karyotypes. Species in Cycadaceae (genus *Cycas*) all have 2*n* = 22 chromosomes with asymmetrical karyotypes characterized by a near equal number of meta (or submetacentric) and telocentric chromosomes. In contrast, the 2*n* = 18 karyotypes of *D. edule*, *D. spinolosum*, and *M. communis* (all Zamiaceae) are more symmetrical, comprised mostly of metacentric or submetacentric chromosomes (e.g., see Table 2 and Table S3). Ancestral chromosome number reconstructions (Figure 5) suggest that the MRCA of all cycads likely had a relatively low number of chromosomes (approximately *n* = 8). However, as Gorelick et al. (2014) have already pointed out, ancestral chromosome number reconstructions must be treated with some caution, particularly when there is a high probability that extinct representatives are missing from the analysis, which seems likely given the analyses of Condamine et al. (2015). It is noteworthy that the large blocks of telomeric‐like repeats in the centromeres of *Cycas* telocentric chromosomes occur in a likely context of genome downsizing as divergence from the MRCA of all cycads (Figure 6). Despite this caution, our analysis does suggest that the predominant direction of chromosome evolution in most cycads is towards increasing numbers, from *n* = 8 to *n* = 9 and *n* = 11 (Figure 5), probably involving centric fissions, and associated with genome downsizing in *Cycas*. Such a process could have been associated with chromosome shuffling reported in other groups of gymnosperms (de Miguel et al., 2015). In the case of most other species, the family Zamiaceae was observed genome upsizing followed by downsizing (Figure 6).

Chromosome number increases associated with polyploidy have been reported in many angiosperm lineages. However, such an explanation is unlikely to explain a hypothesized increase in chromosome number with cycad divergence given the absence of signatures of any ancestral polyploidy in *Cycas* genome sequence as the MRCA of cycads, and there is no evidence of more recent polyploidy apparent in chromosome numbers and resolvable by the ChromEvol program.

In contrast to the Marchant (1968) hypothesis of chromosome divergence in cycads, we find no support for telocentric karyotypes representing the ancestral condition of cycads or of Zamiaceae (the so‐called “prototelocentric hypothesis”). Instead, the ancestral karyotype reconstruction here (Figure 5) suggests that the putative cycad progenitor probably consisted mostly of (sub)metacentric chromosomes, which diverged predominantly by fission events leading to increases in chromosome numbers. This scenario might be different in a clade within Zamiaceae comprising *Zamia variegata*, *Zamia picta*, and *Zamia paucijuga*, where chromosome numbers are predicted to have reduced, indicating that chromosome fusions may have occurred (Figure 5, boxed).

Despite a view that cycads show a slow rate of evolution (Chaw et al., 2008; Gorelick & Olson, 2011), species radiation at genus level are thought to have occurred over the last 19 MYA (Condamine et al., 2015; Nagalingum et al., 2011; Renner, 2011). In *Zamia* this is associated with much karyotype divergence. Of note, *Zamia loddigesii* (syn. *Zamia polymorpha*) exhibits great intraspecific karyotypic diversity (2*n* = 17, 18, 22, 23, 24, 25, 26, 27, and 28 cytotypes) (Table S3), revealing that genome divergence can be highly dynamic in some species. For this cycad in particular, such variation has been attributed to selection pressures associated with habitat (Vovides et al., 2003).

If chromosome fissions do indeed predominantly explain karyotype diversity in cycads, it suggests that pericentromeric telomere sequences, as observed in *Cycas* do not have their origin in ancient chromosome centromeric fusions. It is also thought that telocentric chromosomes generally do not occur (or rarely) in species bearing interstitial and pericentromeric telomeric loci (Fuchs et al., 1995; Mandakova et al., 2015; Mlinarec et al., 2019; Schubert & Rieger, 1990; Sousa & Renner, 2015), which raised doubts about their origin by chromosome fusion/fission and alternative hypotheses were proposed (reviewed in Maravilla et al., 2021).

## CONCLUSION

Although the evolution of karyotypes in cycads has been suggested to be driven by drift rather than selection (Gorelick & Olson, 2011), there might be selection for karyotypes with increased number of chromosomes, which is likely to lead to larger numbers of recombination events per meiotic cycle to generate genetic diversity (because each chromosome is likely to be associated with two or more recombination events). Gorelick (2009) argued there is much linkage disequilibrium in species with few large chromosomes. Thus, fissions leading to increased chromosome numbers, as appears to occur with the radiation of cycads, could be associated with elevated rates of species divergence. Previously, it has been reported that there was a rapid diversification of cycad genera approximately 5–10 MYA followed by a slowdown in diversification and continuing to this day (Nagalingum et al., 2011). The reduced species rates of diversification over the last 10 million years might be explained by an absence of polyploidy, slow reproductive cycles, and strict diocecy, all associated with an ever‐diminishing global abundance.

## EXPERIMENTAL PROCEDURES

### Plant material

The origin of the plant species analyzed, and the methods used for analysis are listed in Table S4. For cytogenetic experiments, seeds of *M. communis, D. spinulosum*, and *C. siamensis* (obtained from a commercial source at neoseeds.cz) were germinated in the local greenhouse (Czech Academy of Science, Brno, Czech Republic) and roots from young (1‐year‐old) plants were collected. *Cycas revoluta* (about 30 cm height) was bought in a commercial shop, Banovce nad Bebravou, Slovakia and grown in a greenhouse. Roots or young leaves from *D. edule, Z. furfuracea*, and *C. circinalis* were collected in the Botanical Garden of Masaryk University (Brno, Czech Republic). *Cycas debaoensis*, *C. multipinnata*, and *C. multifrondis* were from Kunming Institute of Botany (Kunming, China).

### DNA isolation and Illumina sequencing

The sequencing of genomic DNA isolated from 24 cycad, five other gymnosperm, and one angiosperm species listed in Table S4 was carried out by BGI Genomics (BGI, Hong Kong), using an Illumina HiSeq 4000 or DNBseq platforms (<800‐bp library insert, paired end reads, generating 125–150 bp read lengths depending on experiment). Typically, 3–8 Gb data were obtained. FASTQ format sequencing reads were supplied with adapter sequences removed. A summary of the sequencing information and accession numbers of sequence read archives (NCBI, Bioproject PRJNA665617) is available in Table S1.

### Estimation of telomeric repeats abundance and diversity from high‐throughput sequencing data

The abundance of telomeric repeats was determined according to the GP, calculated from the number of mapped reads to total reads (in percentage). The cleaned reads (trimmed for the quality, Phred score >30 over >90% read length, CLC workbench tool, Qiagen, Hilden, Germany) were mapped to the *Arabidopsis* telomere type reference sequence. To optimize the length of the reference sequence we used *N. tabacum* (tobacco), whose telomeric fragments have previously been determined experimentally (Fajkus et al., 1995), and for which several read archives are available (Renny‐Byfield et al., 2012; Sierro et al., 2014). Using the SRR343012 archive of Illumina genomic reads (SR‐1 variety) we obtained a nearly identical number of telomeric reads by using 12–16‐mers containing telomeric sequence motifs. Therefore, in all experiments a 98‐nt long (TTTAGGG)_14_ sequence was chosen as a reference. Mapping to the reverse (CCCTAAA)_14_ sequence showed similar results. Mapping parameters were chosen to map reads containing at least 60% of the telomeric sequence with at least eight consecutive telomeric units. These relatively stringent conditions allowed us to estimate the genomic abundance of longer, relatively homogeneous telomeric repeats, while loci with shorter repeats were not detected. The stringency of alignments was set to 80% allowing, on average, 1.3 mismatches per TTTAGGG heptanucleotide motif. Mapped sequences were evaluated by read coverage graphs. Only files with evenly covered reference sequences were considered for the analysis of GP and sequence diversity. The genome space in Mb was calculated as the proportion of the whole genome according to the formula: GP*genome size (Mb/1C), with genome size values taken from the Plant DNA C‐values database (Pellicer & Leitch, 2020), and in case of missing data from specific papers, e.g., Du et al. (2017).

The sequence diversity of the telomeric repeats was determined by analyzing the variants using the “Variant detection” tool in CLC. The SNVs were called using the following parameters: Read coverage_ >400, Variant count_ >40, and Frequency threshold_ >20%. Reads from mapped files were extracted and searched for the telomeric repeat variants. First, all 21 possible variants differing from the canonical *Arabidopsis*‐type TTTAGGG by a single substitution were included in the motif list. A “Search motif” tool (CLC) was then used to identify telomeric motif variants in reads extracted from mapped files. Only one of the DNA strands was considered. The numbers of individual motifs were counted after filtering that motif. Only motifs occurring at ≥10% frequency were scored. The second less stringent search allowed two substitutions. Through this approach a relatively abundant TTCAAGG variant differing from the canonical *Arabidopsis* type in the third and fifth position was revealed in some genomes. The genome proportion of a particular telomeric variant was expressed as the percentage of that motif versus all 22 variants (including the *Arabidopsis*‐type and the double mutated TTCAAGG) motifs.

The datasets and bioinformatic procedures used to calculate the genome abundance and diversity of telomeric repeats are given in Table S2. The correlation statistics were calculated using tools in msexcel and an online Descriptive statistics software (Wessa, 2017).

### Ancestral chromosome and genome size reconstruction

The evolution of chromosome numbers across cycad species was inferred using ChromEvol v. 2.0 (Glick & Mayrose, 2014) and the Bayesian phylogenetic tree by Condamine et al. (2015). Haploid chromosome numbers (*n*) for 107 species were obtained from the Chromosome Counts Database (CCBD; Rice et al., 2015). In total, eight models of chromosome evolution were tested. ChromEvol assesses the fit of various types of chromosome number change along the phylogenetic tree, inferring the type of transition in chromosome number (duplication, demi‐duplication, and gain and loss of chromosomes), and their likelihood, along the branches of the tree. The Akaike Information Criterion was used for comparing and selecting the best fitting model of chromosome evolution. The output results (i.e., posterior probabilities of ancestral chromosome number and transitions inferred) from this model were plotted in the phylogenetic tree. We also reconstructed ancestral genome sizes using the same phylogenetic tree as for chromosome evolution and the available C‐values from the Plant DNA C‐value database (https://cvalues.science.kew.org/; Pellicer & Leitch, 2020), mostly coming from Ohri and Khoshoo (1986), Zonneveld (2012), and Zonneveld and Lindstrom (2016). The ancestral genome sizes of the MRCA of each clade were estimated with the *fastAnc* function available in the library Phytools (Revell, 2012), which uses a Maximum Likelihood approach for the reconstruction. The results were then plotted onto the phylogenetic tree using the *contMap* function of the same library for visualization, and the reconstructed values depicted at nodes of interest.

### Southern blot hybridization and DNA methylation analysis

Southern blot hybridization was carried out using standard procedures described previously (Kovarik et al., 2005). Genomic DNAs were digested with an excess of *Tru*1I, *Taq*I, and *Hae*III (Takara, Kusacu, Japan) restriction enzymes and the resulting fragments subjected to agarose gel electrophoresis. For methylation analysis the DNA was digested with the McrBC enzyme (20 units) in the reaction mixture supplemented with 1 mm GTP following manufacturer's (New England Biolabs, Ipswich, MA, USA) recommendations. The very short recognition motif (R^m^C, where R is adenine or guanine and ^m^C is 5‐mC) allows detection of a large proportion of the methylated DNA. Probes were hybridized to the blots at 55°C for 16 h in a hybridization oven. Membranes were washed at 55°C with 0.6× sodium citrate/chloride buffer (SSC; 2 × 15 min) followed by 0.2× SSC (2 × 15 min). Membranes were exposed to phosphor screens, scanned with a PhosphorImager (Typhoon FLA9000; GE Healthcare, Bensalem, PA, USA) and the signals analyzed using ImageQuant software (GE Healthcare, Bensalem, PA, USA).

### DNA probe preparation for FISH and southern blotting

For FISH, probes were amplified by non‐template polymerase chain reaction (PCR) using 28 nt oligonucleotide primers. The *Arabidopsis*‐type telomeric primers were: forward: (AGGGTTT)_4_ and reverse: (AAACCCT)_4_. The TTCAGGG variant primers were: forward: (AGGGTTC)_4_ and reverse: (GAACCCT)_4_. The PCR reaction mixture contained 210 μl water, 80 μl 10× KAPA Taq Buffer (Kapabiosystems; Merck KGaA, Darmstadt, Germany), 12 μl 20 mm dNTPs, 6 μl of each 100 μm primer, 6 μl 5 U/μl KAPA Taq polymerase. Non‐template PCR steps were: 5 cycles of 94°C/60 sec, 55°C/30 sec, 72°C/60 sec, 30 cycles of 94°C/60 sec, 60°C/30 sec, 72°C/90 sec, and a final elongation step of 72°C for 5 min. The PCR products were purified by precipitation (1/10 volume 3 m sodium acetate and 2.5 volume 100% ethanol) for 30–60 min at −20°C followed by centrifugation at 12 000 *g* for 1 min. The pellet was washed twice with 70% ethanol, dried at room temperature, and dissolved in the elution buffer (10 mm Tris‐Cl, pH 8.5). The probes were labeled by nick translation using 5‐fluorescein dUTP (Enzo Life Sciences, Farmingdale, NY, USA) for the TTTAGGG repeat, and Cy3‐dUTPs (Roche, Basel, Switzerland) for the TTCAGGG repeat.

For Southern blotting the probes used were 28‐nt oligonucleotides derived from C‐rich strands of TTTAGGG and TTCAGGG telomeric repeats. Oligonucleotides (50 pmol) were labeled at the 5′ terminus with 10 μCi of radioactive [^32^P]‐ATP in a polynucleotide kinase (New England Biolabs, Ipswich, MA, USA) reaction.

### FISH

Root tips were collected and transferred to ice‐cold water and incubated at 4°C overnight. Young leaves were pretreated with 2 mm 8‐hydroxyquinoline at room temperature for 4 h. Both tissues were then fixed in 3:1 (v/v) 100% ethanol/99% glacial acetic acid solution overnight before stored in 70% ethanol at −20°C. For slide preparation a previously published protocol (Wang et al., 2016) was followed with a few modifications: the enzymatic treatment comprised 1% pectinase (SERVA, from *Aspergillus niger*), 2% cellulase Onozuka R‐10 (SERVA, from *Trichoderma viride*) in 1× citrate buffer (4 mm sodium citrate dihydrate, 6 mm citric acid) for 60–90 min at 37°C. Slides were prepared using the “SteamDrop” technique following the protocol of Kirov et al. (2014). Briefly, a cell suspension was vortexed after the enzymatic treatment, 470 μl water was added and the sample was centrifuged at 10 000 *g* for 2 min. The supernatant was washed with 470 μl of 100% ethanol. The pellet was re‐suspended in ethanol (10 μl per slide) and the resulting suspension dropped onto a slide and 20 μl of the first fixation mixture (100% ethanol/99% glacial acetic acid [3:1]) was subsequently added. The slide was inverted upside down under the steam (generated by a water bath heated to 55–60°C) for 15–20 sec and 5 μl of the second fixation solution (100% ethanol/99% glacial acetic acid [2:1]) added. The step was repeated, and the slides dried at room temperature and stored in a fridge at 8–10°C until use.

Before hybridization, slides were treated with 100 μl of 100 μg ml^−1^ RNase A (R6513; Sigma‐Aldrich, Saint‐Louis, MO, USA) per slide, incubated in a humid chamber at 37°C for 20–30 min, washed in 2× SSC (0.3 m sodium chloride and 0.03 m tri‐sodium citrate) for 10 min, post‐fixed in 2% (v/v) formaldehyde in 1× phosphate‐buffered saline (PBS) for 5 min, washed in distilled water for 10 min and dehydrated in an ethanol series (75%, 85%, and 100% ethanol). The individual of *C. circinalis* analyzed had lignified roots that were unsuitable for microscopic preparations. For this species we therefore used young leaf material following the protocol of Kokubugata and Forster (2006).

The hybridization reaction mixture contained 15 μl 100% formamide (F5786 BioUltra, Sigma‐Aldrich, Saint‐Louis, MO, USA), 6 μl 50% dextran sulfate (Sigma‐Aldrich, Saint‐Louis, MO, USA), 3 μl 20× SSC (3 m sodium chloride and 0.3 m tri‐sodium citrate) and 6 μl labeled probes. Optionally, water was added to give a final volume of 30 μl. Probes were denatured at 85°C for 12 min, and incubated on ice for 10–15 min. The probe‐slide hybridization was carried out initially at 80°C for 10 min followed by overnight incubation at 40°C (higher stringency conditions than usually used at 37°C). After hybridization, slides were washed twice in 2× SSC at 42°C, twice in 20% formamide in 0.1× SSC, 4 × 5 min in 2× SSC, 4× SSC in 0.1% Tween 20 for 7 min and briefly rinsed in 1× PBS. The stringency of washes calculated from a formula in Schwarzacher and Heslop‐Harrison (2000) leading to the hybridization of probe and target sequence identities greater or equal to 96.95% and 91.21% for the TTCAGGG and TTTAGGG probes, respectively. Slides were mounted in Vector Shield TM mounting medium containing 4,6‐diamidino‐2‐phenylindole (DAPI) (Vector Laboratories, Newark, CA, USA). The slides were scanned using an epifluorescence microscope (Olympus Provis AX70, Japan) and imaged using Cold Cube Camera 1 (MetaSystems Probes GmbH, Altlussheim, Germany). The imaging software was ISIS (MetaSystems Probes GmbH, Altlussheim, Germany). Images were optimized for contrast and brightness with Adobe Photoshop 2020.

### Immunohistochemical staining of chromosomes

Root tips were harvested from growing young plants and treated with ice‐cold water for 30–60 min, and fixed in 4% paraformaldehyde (Alfa Aesar; Thermo Fisher Scientific, Waltham, MA, USA) in PBS, pH 7.0 on ice for 25 min. Roots were washed in 1× PBS and enzymatically treated at 37°C for 30–45 min (1% pectinase and 2% cellulase Onozuka R‐10 in 1× PBS). Root tips were transferred to 1× PBS and mounted and squashed on poly‐L‐lysine slides (VWR International, Emeryville, CA, USA), the cover slide was removed in liquid nitrogen and rinsed in 1× PBS for 5 min. Slides were incubated in blocking solution 3% bovine serum albumin (BSA; Fraction V; SERVA) in 0.1% Triton X‐100 (Sigma‐Aldrich) for 60 min in a humid chamber at 37°C. Primary antibody against H3S10P (ab5176; Abcam, Cambridge, UK) was dissolved in 1% BSA, 0.1% Tween 20, and a 1:300 dilution applied onto the slide, before incubation for 60 min at 37°C. Slides were then washed with 1× PBS (3 × 5 min) and the second antibody labeled with the FITC fluorochrome (goat antirabbit IgG (H + L), AB‐2337984; Jackson ImmunoResearch, West Grove, PA, USA) was added (1:300 dilution in 1% BSA, 0.1% Tween 20) and incubated for 60 min at 37°C. After washing three times in 1× PBS and dehydrated through an ethanol series (75%, 85%, and 100%), slides were mounted in VectaShield mounting medium containing DAPI and observed in the microscope same as FISH slides, mentioned previously.

For the combined immunochemical detection of 5‐mC and telomere‐FISH, the protocol followed that of standard FISH (see above). Briefly, the hybridization mixture contained 3 μl of 5‐fluorescein dUTP‐labeled TTTAGGG probe. The mixture was denatured at 75°C for 10 min and rapidly cooled on ice. The dry slides were denatured in a hybridization mixture on a hot plate at 80°C for 2 min. The following steps were carried out in the dark. Slides were washed in 2× SSC, 1× PBS (5 min each) and after the addition of 100 μl of blocking solution, slides were incubated in a humid chamber at 37°C for 30 min. The antibody against 5‐mC (ab214727; Abcam) was dissolved in 1% BSA, 0.1% Tween 20 (1:150 dilution), immediately applied to the slides and incubated at 37°C for 60 min. Slides were then washed with 1× PBS (3 × 5 min) followed by the addition of the second antibody labeled with Cy3 (goat antirabbit IgG (H + L); AB‐2313593; Jackson ImmunoResearch) (diluted 1:200 in 1% BSA, 0.1% Tween 20 in 1× PBS) and incubated at 37°C for 60 min. After washing (3 × 5 min in 1× PBS) slides were dehydrated through an ethanol series (75%, 85%, and 100%), mounted in the VectaShield mounting medium with DAPI and observed in a microscope.

## Author CONTRIBUTIONS

The study was conceived by AK, ARL, and IJL. RS and OP. IJL, FS, RV, and WW collected/grew all plant accessions. AK, RV, FS, and WW conducted whole genome sequencing. AK, ARL, JP, and RV analyzed the data. Ancestral karyotype and genome size computation was performed by JP. Cytogenetic work was carried out by RV, JL, and WW. AK and RV conducted bioinformatic analysis and Southern hybridization. AK and ARL wrote the manuscript with inputs from IJL and JP.

## Supporting information


**Table S1**. Details of genome skimming data generated for this study.

**Table S2**. Data used to estimate telomeric repeat abundance and SNV variation.

**Table S3**. Karyotypic characteristics and the genome size of the cycad species analyzed in Figures 5 and 6.

**Table S4**. List of species and the types of analyses conducted.

**Figure S1**. Telomeric repeat abundance in plant genomes expressed in Mb/1C (a) and as a genome proportion in percentages (b).
**Figure S2**. Structural analysis of telomeric repeats in several gymnosperm and angiosperm species by Southern blot hybridization. (a) Terminal telomeric fragments revealed by hybridizing a TTTAGGG probe (inverse complement) to genomic DNAs of six gymnosperms and one angiosperm (*Nicotiana* tabacum) digested with *Tru*1I (Tr), *Taq*I (Ta), and *Hae*III (Ha) restriction enzymes. Note the extensive digestion with *Tru*1I of DNAs containing telomeric repeats in *Cycas revoluta* and *Cycas circinalis* (arrow).
**Figure S3**. DNA methylation analysis of telomeric repeats by methylation‐sensitive restriction enzyme. The genomic DNAs from *Cycas revoluta* and *Nicotiana tabacum* were digested with the McrBC enzyme cutting motifs containing 5‐mC. Hybridization probes were tetrameric oligos composed inverse complements of TTTAGGG (a) and TTCAGGG (b), respectively.
**Figure S4**. Immunohistochemical staining of cycad prophase chromosomes with an antibody to 5‐methylcytosine (5‐mC) and by telomere‐FISH. Note in *Cycas revoluta* that there is an accumulation of 5‐mC signals overlapping with blocks of telomeric repeats (a). Metaphase, prophase or anaphase chromosomes of *Dioon edule* (b), *Macrozamia communis* (c), and *Zamia furfuracea* (d), respectively, show 5‐mC signals with several local maxima and minima. In these three species the telomere‐FISH was not provided. Bar = 10 μm.
**Figure S5**. Telomeric FISH showing the location of hybridization signals from two different telomeric probes separately for five cycad species. There were 11 and 12 strong telomeric probe signals in *Cycas revoluta* male (a) and female (b), respectively. Distribution of telomere probes TTTAGGG (green), and TTCAGGG (red) on chromosomes of *Cycas circinalis* (c), *Cycas siamensis* (d), *Dioon edule* (e) and *Macrozamia communis* (f). Bar = 10 μm.
**Figure S6**. Telomeric FISH to *Cycas multipinnata* metaphase (a) and interphase (b,c). Note the fusion of telomeric blocks in (a) and the tendency for telomeric blocks to cluster in interphase (b,c). Hybridization *s*ignals in telomeric positions are barely visible probably due to low copy numbers of repeats and/or size differences between the chromosomes.
**Figure S7**. Telomeric FISH to *Cycas debaoensis* (a), *Cycas multifrondis* (b), and *Zamia furfuracea* (c). In (a) note the strong and weak probe hybridization to proximal regions of telocentric and (sub)metacentric chromosomes, respectively. Hybridization *s*ignals in telomeric positions are barely visible probably due to the low copy number of repeats and/or size differences between the chromosomes, bar = 10 μm.
**Figure S8**. Phylogenetic trees showing very different levels of homogeneity in telomeric arrays between *Cycas revoluta* and *Nicotiana tabacum*. The trees were constructed from 300 aligned sequences. The sequences were obtained by a BLAST search of stand‐alone databases of telomeric reads (obtained from mapping files). A 5‐mer of the *Arabidopsis* repeat was used as the query.

**Data S1**. Example of randomly selected reads from *Cycas revoluta* with highlighted telomeric variants. Reads were obtained by mapping of Illumina genomic reads to the *Arabidopsis* 14‐mer reference sequence.

## Data Availability

The sequence data have been deposited in the NCBI Sequence Read Archive (BioProjects ID PRJNA665617 and PRJNA634996), and the files are available under the SRA studies SRP285716 (Chromosome evolution in ancient gymnosperm species) and SRP265576 (*Tragopogon porrifolius* subsp. *porrifolius* genome sequencing).
