# Supplementary material for: Mega‐sized pericentromeric blocks of simple telomeric repeats and their variants reveal patterns of chromosome evolution in ancient Cycadales genomes
Source: Plant J. 2022 Oct 11;112(3):646–63. doi: 10.1111/tpj.15969 (PMC9827991; doi:10.1111/tpj.15969)
Supplement: Supplementary file 1 — Table S1. Details of genome skimming data generated for this study. [file TPJ-112-646-s006.pdf]

Table S1. Details of genome skimming projects carried out within this study.

Species	ID/sampling	Collection number [a]	Sequencing platform/Company [b]	No. of read pairs [c]	SRA accession number (NCBI)
Cycas changjiangensis	2018/SCBG,CN	SCBG: ww1805hny_010	Illumina Hiseq4000/ BGI	8955387	SRR12737687
Cycas circinalis	2014/MU,Brno,CZ	MU: ak_2014cc_1	Illumina Hiseq2500/Eurofins	29967062	SRR12737686
Cycas diannanensis	2018/KIB,CN	KIB: ww1804km_033	Illumina Hiseq4000/ BGI	8961696	SRR12737675
Cycas guizhouensis	2018/KIB,CN	KIB-ww1804km_031	Illumina Hiseq4000/ BGI	8962134	SRR12737667
Cycas hainanensis	2018/SCBG,CN	SCBG: ww1805hny_006	Illumina Hiseq4000/ BGI	8956276	SRR12737666
Cycas media	2018/Kew,UK	Kew: 1987-8113	Illumina Hiseq4000/ BGI	7712742	SRR12737665
Cycas panzhihuaensis	2018/KIB,CN	KIB: ww1804km_030	Illumina Hiseq4000/ BGI	8961084	SRR12737664
Cycas pectinata	2018/Kew,UK	Kew: 1978-340	Illumina Hiseq4000/ BGI	8954628	SRR12737663
Cycas revoluta	2018/CPG,UK	CPG: 1990-0350/1	Illumina Hiseq4000/ BGI	8954260	SRR12737662
Cycas rumphii	2018/Kew,UK	Kew: 1966-59801	Illumina Hiseq4000/ BGI	19181408	SRR12737661
Cycas sexseminifera	2018/KIB,CN	KIB: ww1804km_034	Illumina Hiseq4000/ BGI	8952297	SRR12737685
Cycas siamensis	2018/Kew,UK	Kew: 1969-17789	Illumina Hiseq4000/ BGI	16538222	SRR12737684
Cycas taitungensis	2018/Kew,UK	Kew: 1981-8688	Illumina Hiseq4000/ BGI	19174471	SRR12737683
Cycas thouarsii	2018/Kew,UK	Kew: 1969-17791	Illumina Hiseq4000/ BGI	20623053	SRR12737682
Ceratozamia hildae	2018/Kew,UK	Kew: 1995-2379	Illumina Hiseq4000/ BGI	21132347	SRR12737677
Dioon edule	2014/MU,Brno,CZ	MU: ak_2014de_1	Illumina Hiseq2500/Eurofins	24556729	SRR12737681
Dioon spinulosum	2020/IBP,Brno,CZ	Commercial source, CZ	DNBSEQ PE150/BGI	25863114	SRR18671860
Encephalartos ferox	2018/SCBG,CN	SCBG: ww1805hny_008	Illumina Hiseq4000/ BGI	8961374	SRR12737679
Encephalartos manikensis	2018/SCBG,CN	SCBG: ww1805hny_007	Illumina Hiseq4000/ BGI	8964756	SRR12737678
Macrozamia communis	2020/IBP,Brno,CZ	Commercial source, CZ	DNBSEQ PE150/BGI	24221756	SRR18671861
Macrozamia moorei	2018/Kew,UK	Kew: 1988-4333	Illumina Hiseq4000/ BGI	21493798	SRR12737676
Stangeria eriopus	2018/Kew,UK	Kew: 1993-1808	Illumina Hiseq2500/Eurofins	26294724	SRR12737680
Zamia fisheri	2018/SCBG,CN	SCBG: ww1805hny_019	Illumina Hiseq4000/ BGI	8955907	SRR12737674
Zamina furfuracea	2018/SCBG,CN	SCBG: ww1805hny_014	Illumina Hiseq4000/ BGI	8960422	SRR12737673
Metasequoia glyptostroboides	2018/UCM,CN	GZUCM: ww1805gzy_003	Illumina Hiseq4000/ BGI	8952940	SRR12737671
Podocarpus macrophyllus	2018/UCM,CN	GZUCM: ww1805yp_001	Illumina Hiseq4000/ BGI	8419865	SRR12737670
Prumnopitys andina	2018/Kew,UK	Kew: 1979-4183	Illumina Hiseq4000/ BGI	7937205	SRR12737669
Retrophyllum minus	2018/Kew,UK	Kew: 2013-1495	Illumina Hiseq4000/ BGI	8421032	SRR12737668
Wollemia nobilis	2018/Kew,UK	Kew: 2009-1665	Illumina Hiseq4000/ BGI	7215484	SRR12737672
Tragopogon mirus	2014/2602/IBP,Brno,CZ	Brno: rm2602	Illumina Hiseq4000/ BGI	7250058	SRR12710425

Footnotes

[a] Source of the plant material:

SCBG: South China Botanical Garden. China
KIB: Kunming Institute of Botany, China
GZUCM: Guangzhou University of Chinese Medicine, China
Kew: Royal Botanical Garden, Richmond. United Kingdom
MU: Botanical Garden, Masaryk University, Brno, Czech Rebublic
IBP: Institute of Biophysics Czech Academy of Sciences, Brno, Czech Republic

[b] Small fragment library, PCR free.

[c] Total number of read pairs after adaptor trimming and low quality reads removal.
