# Supplementary material for: Mega‐sized pericentromeric blocks of simple telomeric repeats and their variants reveal patterns of chromosome evolution in ancient Cycadales genomes
Source: Plant J. 2022 Oct 11;112(3):646–63. doi: 10.1111/tpj.15969 (PMC9827991; doi:10.1111/tpj.15969)
Supplement: Supplementary file 2 — Table S2. Data used to estimate telomeric repeat abundance and SNV variation. [file TPJ-112-646-s001.pdf]

Table S2. Data used to estimate telomeric repeat abundance and SNV variation

Group	*Species*	Number of input reads	Number of mapped reads	Genome proportion [%]	Genome size [Gb/1C][a]	Telomere sequences [Mb/1C]	Number of telomeric units [copies per 1C]	SNV (number)
Cycadaceae	*Cycas circinalis*	29 967 062	49 998	0.1668	13.14	21.92	3 131 885	44
	Cycas diannanensis	8 961 696	10 020	0.1118	13.10	14.65	2 092 429	28
	Cycas guizhouensis	8 962 134	6 071	0.0677	12.70	8.60	1 229 007	48
	Cycas hainanensis	8 956 276	147 293	1.6446	13.10	215.44	30 777 114	48
	Cycas changjiangensis	8 955 387	196 109	2.1898	12.70	278.11	39 730 026	48
	Cycas media	7 712 742	126 982	1.6464	13.20	217.32	31 046 257	48
	Cycas panzhiuhuanensis	8 961 084	33 496	0.3738	13.10	48.97	6 995 289	34
	Cycas pectinata	8 954 628	87 137	0.9731	13.10	127.48	18 210 770	48
	Cycas revoluta /RBG Kew, UK/	7 098 576	81 077	1.1422	13.40	153.05	21 864 180	44
	Cycas revoluta /Kunming IB, CN/	8 954 260	121 110	1.3525	13.40	181.24	25 891 491	46
	Cycas rumphii	19 181 408	17 603	0.0918	15.10	13.86	1 979 635	42
	Cycas sexseminifera	8 952 297	100 001	1.1170	14.40	160.85	22 979 169	48
	Cycas siamensis	16 538 222	295 928	1.7894	13.10	234.41	33 486 557	48
	Cycas taitugensis	19 174 471	275 585	1.4372	13.10	188.28	26 897 099	48
	Cycas thouarsii	20 623 053	48 681	0.2361	12.70	29.98	4 282 646	48
subtotal	*15*							
Zamiaceae	*Ceratozamia hildae*	21 132 347	44 174	0.2090	30.99	64.78	9 254 278	10
	Dioon edule	24 556 729	436	0.0018	24.60	0.44	62 395	0
	Dioon spinulosum	25 836 114	134	0.0005	24.26	0.13	17975	0
	Encephalartos ferox	8 961 374	17 461	0.1948	30.74	59.90	8 556 582	16
	Encephalartos manikensis	8 964 756	22 514	0.2511	28.52	71.62	10 232 121	12
	Macrozamia communis	24 221 756	127	0.0005	26.17	0.14	19 602	0
	Macrozamia moorei	21 493 798	2 067	0.0096	25.20	2.42	346 202	24
	Stangeria eriopus	26 294 724	305	0.0012	14.50	0.17	24 027	0
	Zamia fisheri	8 955 907	3 864	0.0431	18.28	7.89	1 126 693	24
	Zamia furfuracea	8 960 422	5 699	0.0636	17.79	11.31	1 616 397	24
subtotal	*10*							
Other gymnosperms	*Abies sibirica*	15 736 945	1 108	0.0070	15.48	1.09	155 701	0
	Ephedra altissima	10 071 542	700	0.0070	30.00	2.09	297 869	4
	Ginkgo biloba	37 581 985	960	0.0026	11.50	0.29	41 965	0
	Gnetum gnemon	8 655 982	2 819	0.0326	3.79	1.23	176 327	0
	Gnetum montanum	11 158 841	2 004	0.0180	4.20	0.75	107 753	0
	Juniperus cedrus	46 193 335	2 006	0.0043	11.20	0.49	69 482	0
	Metasquoia glyptostroboides	8 952 940	2 251	0.0251	10.82	2.72	388 632	2
	Picea glauca	1 826 244	33	0.0018	15.83	0.29	40 864	0

	Pinus taeda	37 720 440	10 364	0.0275	22.60	6.21	887 076	0
	Podocarpus macrophyllus	8 419 865	3 953	0.0469	9.50	4.46	637 158	4
	Prumnopitys andina [b]	7 937 205	2 929	0.0369	6.92	2.55	364 804	0
	Retrophyllum minus [b]	8 421 032	5 970	0.0709	5.78	4.10	585 381	24
	Taxus bacata	38 689 870	43 698	0.1129	10.83	12	1 747 410	0
	Welwitschia mirabilis	3 748 384	229	0.0061	7.06	0.43	61 617	0
	Wollemia nobilis	7 215 484	418	0.0058	13.66	0.79	113 048	0
subtotal	*15*							
Angiosperms	*Amborella trichopoda*	17 331 748	27 758	0.1602	0.87	1.39	199 052	0
	Anacyclus pyrethrum [b]	2 451 902	3 682	0.1502	5.78	8.68	1 239 968	0
	Anacyclus radiatus	15 862 528	7 273	0.0459	8.33	3.82	545 617	4
	Arabidopsis thaliana	6 193 672	3 044	0.0491	0.16	0.08	10 953	0
	Arachis hypogeae	18 557 082	6 948	0.0374	2.81	1.05	150 407	0
	Asparagus officinalis	4 433 063	2 148	0.0485	1.43	0.69	98 985	0
	Ballantinia antipoda	4 766 725	74 484	1.5626	0.47	7.34	1 049 162	14
	Beta vulgaris	1 989 909	4 216	0.2119	0.9	1.91	272 403	0
	Brassica napus	7 106 431	3 919	0.0551	1.08	0.60	85 084	0
	Cardamine amara	8 032 873	892	0.0111	0.237	0.03	3 760	0
	Cucumis pepo	12 080 110	18 212	0.1508	1.05	1.58	226 140	0
	Fritillaria imperialis	32 967 735	1 913	0.0058	42.50	2.47	352 303	0
	Genlisea nigrocaulis	12 452 164	3 771	0.0303	0.086	0.03	3 721	0
	Gossypium hirsutum	34 740 690	3 238	0.0093	2.35	0.22	31 290	0
	Hordeum vulgare	2 729 987	1 338	0.0490	5.39	2.64	377 386	0
	Chenopodium quinoa	43 294 938	3 502	0.0081	1.45	0.12	16 755	0
	Lathyrus latifolius	6 630 845	392	0.0059	8.39	0.50	70 857	0
	Lilium tsingtauense	20 473 388	1 187	0.0058	71.18	4.13	590 000	0
	Nicotaina tabacum	11 114 899	8 861	0.0797	5	3.99	569 442	0
	Nicotiana sylvestris	7 479 035	2 088	0.0279	2.5	0.70	99 707	0
	Nicotiana tomentosifomis	6 742 625	7 057	0.1047	2.5	2.62	373 795	0
	Oryza sativa	1 989 909	4 216	0.2119	0.49	1.04	148 308	0
	Quercus rubor	7 350 058	2 900	0.0395	0.8	0.32	45 092	0
	Rosa canina	28 495 260	1 555	0.0055	1.3	0.07	10 135	0
	Secale cereale	20 173 875	923	0.0046	8.6	0.39	56 210	0
	Tanacetum cinerariifolium	8 495 303	1 585	0.0187	7.1	1.32	189 239	2
	Tragopogon mirus	7 350 058	2 900	0.0395	5.12	2.02	288 589	0
	Vicia faba	7 125 880	1 457	0.0204	13	2.66	379 723	0
	Zea mays	354 116	94	0.0265	2.65	0.70	100 492	0
subtotal	*29*							
	Anthoceros punctuatus	400 000	44	0.0110	0.18	0.02	2 829	0
Bryophyta	*Apopellia endiviifolia*	30 523 713	463	0.0015	3.37	0.05	7 303	0
	Dicranum scoparium	7 461 840	526	0.0070	0.71	0.05	7 150	0

	Lunularia cruciata	735 610	39	0.0053	0.65	0.03	4 923	0
	Marchantia polymorpha	500 000	42	0.0084	0.29	0.02	3 480	0
	Pallavicinia lyelli	693 366	128	0.0185	7.98	1.47	210 452	0
	Physcomitrella patens	26 918 967	9 271	0.0344	0.52	0.18	25 584	0
	Polytrichum formosum	50 084 883	2 237	0.0045	0.52	0.02	3 318	0
subtotal	*8*							
**Total**	***77***							

[a]The genome size values were taken from the Plant C value database except Lilium tsingtauense which was according to Du et al. 2017: Front. Plant Sci., 26 https://doi.org/10.3389/fpls.2017.01303

[b]Genome size is not available, the C-value represents the genus average
