# Supplementary material for: Mega‐sized pericentromeric blocks of simple telomeric repeats and their variants reveal patterns of chromosome evolution in ancient Cycadales genomes
Source: Plant J. 2022 Oct 11;112(3):646–63. doi: 10.1111/tpj.15969 (PMC9827991; doi:10.1111/tpj.15969)
Supplement: Supplementary file 3 — Table S3. Karyotypic characteristics and the genome size of the cycad species analyzed in Figures 5 and 6. [file TPJ-112-646-s004.pdf]

Table S3. Karyotypic characteristics and the genome size of the cycad species analyzed in Figure 7 and Table 1.

Family	Genus	Species	T chromosomes	2n	Ratio (T/2n)	1C (Mbp)[a]	Data source (code and the reference)[b]	
**Cycadaceae**	*Cycas*	*C. cairnsiana*	12	22	0.545	n.d.	a	Kokubugata and Forster, 2006
		C. basaltica	12	22	0.545	n.d.	b	Kokubugata and Kondo, 1996
		C. circinalis	12	22	0.545	14455	a	Kokubugata and Forster, 2006
		C. couttsiana	12	22	0.545	n.d.	a	Kokubugata and Forster, 2006
		C. diannanensis	12	22	0.545	12838	a	Kokubugata and Forster, 2006
		C. media	12	22	0.545	13107	a	Kokubugata and Forster, 2006
		C. megacarpa	12	22	0.545	n.d.	a	Kokubugata and Forster, 2006
		C. micronesica	12	22	0.545	n.d.	a	Kokubugata and Forster, 2006
		C. micholitzii	12	22	0.545	13524	a	Kokubugata and Forster, 2006
		C. ophiolitica	12	22	0.545	n.d.	a	Kokubugata and Forster, 2006
		C. revoluta	12	22	0.545	13426	a	Kokubugata and Forster, 2006
		C. rumphii	12	22	0.545	13213	c	Kondo et al. 1995
		C. siamensis	12	22	0.545	13105	a	Kokubugata and Forster, 2006
		C. taitungensis	12	22	0.545	13291	a	Kokubugata and Forster, 2006
		C. taiwaniana	12	22	0.545	n.d.	c	Kondo et al. 1995
		C. thouarsii	12	22	0.545	13230	a	Kokubugata and Forster, 2006
		C. wadei	12	22	0.545	n.d.	a	Kokubugata and Forster, 2006
**Zamiaceae**	*Dioon*	*D. califanoi*	2	18	0.111	n.d.	d	Moretti 1990
		D. caputoi	2	18	0.111	n.d.	d	Moretti 1990
		D. edule	2	18	0.111	24647	d	Moretti 1990
		D. holmgrenii	2	18	0.111	n.d.	d	Moretti 1990
		D. mejiae	2	18	0.111	n.d.	d	Moretti 1990
		D. merolae	2	18	0.111	23863	d	Moretti 1990
		D. purpusii	2	18	0.111	n.d.	d	Moretti 1990
		D. rzedowskii	2	18	0.111	n.d.	d	Moretti 1990
		D. spinulosum	2	18	0.111	n.d.	d	Moretti 1990
		D. tomasellii	2	18	0.111	n.d.	d	Moretti 1990
	Bowenia	*B. serrulata*	0	18	0.000	12524	e	Kokubugata et al. 2001
		B. spectabilis	0	18	0.000	20286	e	Kokubugata et al. 2001
	Stangeria	*S. eriopus*	2	16	0.125	19110	f	Kokubugata et al. 2004
		S. paradoxa	2	16	0.125	n.d.	g	Sax and Beal 1934
	Lepidozamia	*L. hopei*	2	18	0.111	29547	h	Marchant 1968
		L. peroffskyana	2	18	0.111	27097	h	Marchant 1968
	Macrozamia	*M. communis*	2	18	0.111	n.d.	h	Marchant 1968

	M. diplomera	2	18	0.111	n.d.	i	Moretti 1981-82
	M. fawcettii	2	18	0.111	n.d.	h	Marchant 1968
	M. heteromera	2	18	0.111	n.d.	i	Moretti 1981-82
	M. lucida	2	18	0.111	n.d.	h	Marchant 1968
	M. macdonnellii	2	18	0.111	n.d.	h	Marchant 1968
	M. miquelii	2	18	0.111	n.d.	i	Moretti 1981-82
	M. moorei	2	18	0.111	25970	i	Moretti 1981-82
	M. pauli-guilielmi	2	18	0.111	n.d.	h	Marchant 1968
	M. riedlei	2	18	0.111	n.d.	i	Moretti 1981-82
	M. secunda	2	18	0.111	n.d.	i	Moretti 1981-82
	M. spiralis	2	18	0.111	n.d.	i	Moretti 1981-82
	M. stenomera	2	18	0.111	n.d.	h	Marchant 1968
	M. tridentata	2	18	0.111	n.d.	g	Sax and Beal 1934
Microcycas	*M. calocoma*	20	26	0.769	20188	j	Kokubugata and Kondo 1998
Ceratozamia	*C. hildae*	2	16	0.125	n.d.	f	Kokubugata et al. 2004
	C. kuesteriana	2	16	0.125	n.d.	f	Kokubugata et al. 2004
	C. latifolia	2	16	0.125	n.d.	d	Moretti 1990
	C. matudae	2	16	0.125	n.d.	d	Moretti 1990
	C. mexicana	2	16	0.125	31017	f	Kokubugata et al. 2004
	C. miqueliana	2	16	0.125	n.d.	d	Moretti 1990
	C. morettii	2	16	0.125	n.d.	k	Vazquez-Torres et al. 1998
	C. norstogii	2	16	0.125	30968	f	Kokubugata et al. 2004
	C. robusta	2	16	0.125	n.d.	d	Moretti 1990
	C. sabatoi	2	16	0.125	n.d.	l	Vovides et al. 1993
	C. zaragozae	2	16	0.125	n.d.	d	Moretti 1990
Encephalartos	*E. altenseinii*	0	18	0.000	28763	h	Marchant 1968
	E. barteri	0	18	0.000	n.d.	h	Marchant 1968
	E. bubalinus	0	18	0.000	n.d.	h	Marchant 1968
	E. caffer	0	18	0.000	n.d.	m	Mogford 1979
	E. cycadifolius	0	18	0.000	n.d.	h	Marchant 1968
	E. eximius	0	18	0.000	n.d.	h	Marchant 1968
	E. hildebrandtii	0	18	0.000	n.d.	h	Marchant 1968
	E. horridus	0	18	0.000	28420	h	Marchant 1968
	E. humilis	0	18	0.000	n.d.	h	Marchant 1968
	E. lehmannii	0	18	0.000	29155	h	Marchant 1968
	E. manikensis	0	18	0.000	28518	h	Marchant 1968
	E. umbeluziensis	0	18	0.000	n.d.	h	Marchant 1968

	E. villosus	0	18	0.000	20678	h	Marchant 1968
Zamia	*Z. amblyphyllidia*	0	16	0.000	n.d.	d	Moretti 1990
	Z. angustifolia	0	16	0.000	19208	n	Tagashira and Kondo 2001
	Z. cremnophila	0	16	0.000	17591	d	Moretti 1990
	Z. cunaria	14	23	0.609	20923	o	Caputo et al. 1996
	Z. cunaria	16	24	0.667	n.d.	o	Caputo et al. 1996
	Z. fischeri	0	16	0.000	18277	p	Moretti et al. 1991
	Z. fischeri	4	18	0.222	n.d.	p	Moretti et al. 1991
	Z. floridiana	0	16	0.000	n.d.	h	Marchant 1968
	Z. furfuracea	4	18	0.222	17787	n	Tagashira and Kondo 2001
	Z. chigua	12	22	0.545	21315	q	Norstog 1981
	Z. chigua	16	24	0.667	n.d.	q	Norstog 1981
	Z. chigua	18	25	0.720	n.d.	q	Norstog 1981
	Z. chigua	20	26	0.769	n.d.	q	Norstog 1981
	Z. inermis	0	16	0.000	17101	r	Vovides 1983
	Z. integrifolia	0	16	0.000	18718	n	Tagashira and Kondo 2001
	Z. ipetiensis	14	23	0.609	20090	o	Caputo et al. 1996
	Z. loddigesii	6	18	0.333	17493	n	Tagashira and Kondo 2001
	Z. loddigesii	4	18	0.222	n.d.	d	Moretti 1990
	Z. loddigesii	2	17	0.118	n.d.	v	Vovides et al. 2003
	Z. loddigesii	19	27	0.704	n.d.	s	Vovides and Olivares 1996
	Z. loddigesii	7	24	0.292	n.d.	s	Vovides and Olivares 1996
	Z. loddigesii	14	24	0.583	n.d.	s	Vovides and Olivares 1996
	Z. loddigesii	10	24	0.417	n.d.	s	Vovides and Olivares 1996
	Z. loddigesii	11	26	0.423	n.d.	s	Vovides and Olivares 1996
	Z. loddigesii	15	24	0.625	n.d.	s	Vovides and Olivares 1996
	Z. loddigesii	14	25	0.560	n.d.	s	Vovides and Olivares 1996
	Z. loddigesii	15	26	0.577	n.d.	s	Vovides and Olivares 1996
	Z. loddigesii	15	25	0.600	n.d.	s	Vovides and Olivares 1996
	Z. loddigesii	9	25	0.360	n.d.	s	Vovides and Olivares 1996
	Z. loddigesii	12	22	0.545	n.d.	u	Napolitano et al. 2004
	Z. loddigesii	14	23	0.609	n.d.	u	Napolitano et al. 2004
	Z. loddigesii	18	25	0.720	n.d.	u	Napolitano et al. 2004
	Z. loddigesii	20	26	0.769	n.d.	u	Napolitano et al. 2004
	Z. loddigesii	22	27	0.815	n.d.	u	Napolitano et al. 2004
	Z. loddigesii	24	28	0.857	n.d.	u	Napolitano et al. 2004
	Z. loddigesii	5	24	0.208	n.d.	v	Vovides et al. 2003

Z. loddigesii	16	24	0.667	n.d.	u	Napolitano et al. 2004
Z. loddigesii	16	25	0.640	n.d.	v	Vovides et al. 2003
Z. manicata	4	18	0.222	21021	o	Caputo et al. 1996
Z. muricata	20	24	0.833	22393	t	Norstog 1980
Z. muricata	16	23	0.696	n.d.	n	Tagashira and Kondo 2001
Z. obliqua	4	18	0.222	19551	t	Norstog 1980
Z. paucijuga	14	23	0.609	17934	d	Moretti 1990
Z. paucijuga	16	24	0.667	n.d.	d	Moretti 1990
Z. paucijuga	18	25	0.720	n.d.	d	Moretti 1990
Z. paucijuga	20	26	0.769	n.d.	d	Moretti 1990
Z. paucijuga	22	27	0.815	n.d.	d	Moretti 1990
Z. paucijuga	24	28	0.857	n.d.	d	Moretti 1990
Z. paucijuga	6	19	0.316	n.d.	u	Napolitano et al. 2004
Z. paucijuga	14	23	0.609	n.d.	u	Napolitano et al. 2004
Z. paucijuga	18	25	0.720	n.d.	u	Napolitano et al. 2004
Z. paucijuga	22	27	0.815	n.d.	u	Napolitano et al. 2004
Z. paucijuga	24	28	0.857	n.d.	u	Napolitano et al. 2004
Z. picta	10	21	0.476	n.d.	p	Moretti et al. 1991
Z. picta	12	22	0.545	n.d.	p	Moretti et al. 1991
Z. portoricensis	0	16	0.000	n.d.	t	Norstog 1980
Z. pseudomonticola	2	16	0.125	21217	t	Norstog 1980
Z. pumila	0	16	0.000	18571	n	Tagashira and Kondo 2001
Z. purpurea	0	16	0.000	17199	r	Vovides 1983
Z. pygmaea	0	16	0.000	20041	n	Tagashira and Kondo 2001
Z. skinneri	2	18	0.111	19649	n	Tagashira and Kondo 2001
Z. soconuscensis	0	16	0.000	17101	w	Schutzman et al. 1988
Z. spartea	4	18	0.222	17395	s	Vovides and Olivares 1996
Z. tuerckheimii	2	16	0.125	16513	q	Norstog 1990
Z. vazquezii	6	18	0.333	17444	n	Tagashira and Kondo 2001

Legend:

[a] taken from C-value Database cvalues.science.kew.org, n.d. - not determined

[b]

a Kokubugata G., Forster P.I. 2006. Molecular-cytotaxonomy of *Cycas* (Cycadales) Using 5S Ribosomal DNA Markers. National Science Museum Monographs, (34): 163-170.

b Kokubugata G., Kondo K. 1996. Differential fluorescent-banding patterns in chromosomes of four species of *Cycas* (Cycadaceae). Botanical Journal of the Linnean Society. 120:51-55.

c Kondo K., Kokubugata G., Hizume M., Tanaka R., Satake T. 1995. A Karyomorphological Study of Five Species and One Variety of *Cycas.* Cytologia 60:141-147.

d Moretti A. 1990. Karyotypic Data on North and Central American Zamiaceae (Cycadales) and Their Phylogenetic Implications. Amer. J. Bot. 77(8):1016-1029

e Kokubugata G., Hill K.D., Wilson W., Kondo K., Randall L.M. 2001. A comparison of chromosome number and karyotype in somatic chromosomes of *Stangeriaceae* (Cycadales). Edinb. J.Bot. 58(3):475-481.

f Kokubugata G., Vovides A.P., Kondo K. 2004. Mapping 5S ribosomal DNA on somatic chromosomes of four species of *Ceratozamia* and *Stangeria eriopus* (Cycadales). Botanical Journal of the Linnean Society (145): 499-504.

g Sax K., Beal J.M. 1934. Chromosomes of the Cycadales. Journal of the Arnold Arboretum, Vol. 15, No.3: 255-258

h Marchant C.J. 1968. Chromosome Patterns and Nuclear Phenomena in the Cycad Families Stangeriaceae and Zamiaceae. Chromosoma (Berl.) 24: 100-134.

i Moretti A. 1981-1982. Quinacrine fluorescence analysis of the chromosomes of *Macrozamia Miq.* (Cycadales, Zamiaceae). Delpinoa n.c. 23-24:129-136.

j Kokubugata G., Kondo K. 1998. Comparative karyotype analysis of *Ceratozamia mexicana* and *Microcycas calocoma* (Zamiaceae) using fluorochrome banding (CMA/DAPI) and fluorescence in situ hybridizationnn of ribosomal DNA. Pl.Syst. Evol 210: 41-50.

k Vazquez-Torres M., Vovides A.P. 1998. A New Species of Ceratozamia (Zamiaceae) from Veracruz, Mexico. Novon. Spring Vol. 8, No. 1: 87-90.

l Vovides A.P., Vazquez-Torrens M., Schutzman B., Iglesias C.G. 1993. A New Species of Ceratozamia (Zamicaeae) from Querétaro and Hildago, Mexico. Novon. Winter Vol. 3, No. 4: 502-506.

m Mogford D.J. 1979. Heterochromatin in Encephalartos. Cytologia 44:951-954.

n Tagashira N., Konfo K. 2001. Chromosome phylogeny of *Zamia* and *Ceratozamia* by means Robertsonian changes detected by fluorescence in situ hybridization (FISH) technique of rDNA. Plant Syst. Evol. 227:145-155

o Caputo P., Cozzolino S., Gaudio L., Moretti A., Stevenson D.W. 1996. Karyology and Phylogeny of some Mesoamerican species of Zamia (Zamiaceae). American Journal of Botany 83(11):1513-1520

p Moretti A., Caputo P. Gaudio L., Stevenson D.W. 1991. Intaspecific chromosome variation in Zamia (Zamiaceae, Cycadales). Caryologia, (44) 1:1-10.

q Norstog K. 1980. Chromosome Numbers in Zamia (Cycadales). Caryologia: International Journal of Cytology, Cytosystematics and Cytogenetics, 33:3,419-428, DOI:10.1080./00087114.1980.10796855

r Vovides A.P. 1983. Systemaic studies on the Mexicam Zamiaceae. I. Chromosome numbers and karyotypes.1983. Ammer. J. Bot. 70(7):1002-1006

s Vovides A.P., Olivares M. 1996. Karyotype polymorphism in the cycad *Zamia loddigesii* (Zamiaceae) of the Yucatan Peninsula, Mexico. Bot. J. of the Linnean Society. 120:77-83

t Norstog K. 1980. Chromosome Numbers in Zamia (Cycadales). Caryologia: International Journal of Cytology, Cytosystematics and Cytogenetics, 33:3,419-428, DOI:10.1080./00087114.1980.10796855

u Napolitano A., Caputo P., Moretti A. 2004. Karyology, phytogeography, and the origin of intraspecific karyotypic variation in *Zamia paucijuga* and *Z. polymorpha* (Zamiaceae). Delpinoa n.s. 46:71-83.

v Vovides A.P., Perez-Farrera M.A., Gonzales-Astorga J., Gonzalez D., Gregory T., Chemnick J., Iglesias C., Octavio-Aguilar P., Avendano S., Barcenas C., Salas-Morales S. 2003. An outline of our current knowledge on Mexican cycads (Zamiaceae, Cycadales). Plant Biology Vol.4, 159-174

w Schutzman B., Vovides A.P., Dehgan B. 1988. Two new species of *Zamia* (Zamiceae, Cycadales) from Southern Mexico. Bot. Gaz. 149(3):347-360.
