# Supplementary material for: Mega‐sized pericentromeric blocks of simple telomeric repeats and their variants reveal patterns of chromosome evolution in ancient Cycadales genomes
Source: Plant J. 2022 Oct 11;112(3):646–63. doi: 10.1111/tpj.15969 (PMC9827991; doi:10.1111/tpj.15969)
Supplement: Supplementary file 4 — Table S4. List of species and the types of analyses conducted. [file TPJ-112-646-s002.pdf]

Table S4. List of species and the types of analyses conducted.

| Group | Species | Family | Section | SRA accession number [a] | Source | Analysis [b] |
|---|---|---|---|---|---|---|
| Cycadales | *Cycas changjiangensis* | Cycadaceae | Stangerioides | **SRR12737687** | South China Botanical Garden. CN | GEN |
| | *Cycas circinalis* | Cycadaceae | Cycas | **SRR12737686** | Botanical garden Brno, CZ | GEN, FISH, SB |
| | *Cycas debaonensis* | Cycadaceae | Stangerioides | n.a. | Kunming Institute of Botany, CN | FISH |
| | *Cycas diannanensis* | Cycadaceae | Stangerioides | **SRR12737675** | Kunming Institute of Botany, CN | GEN |
| | *Cycas guizhouensis* | Cycadaceae | Stangerioides | **SRR12737667** | Kunming Institute of Botany, CN | GEN |
| | *Cycas hainanensis* | Cycadaceae | Stangerioides | **SRR12737666** | South China Botanical Garden. CN | GEN |
| | *Cycas media* | Cycadaceae | Cycas | **SRR12737665** | Royal Botanical Garden, Kew, UK | GEN |
| | *Cycas multifrondis* | Cycadaceae | Stangerioides | n.a. | Kunming Institute of Botany, CN | FISH |
| | *Cycas multipinnata* | Cycadaceae | Stangerioides | n.a. | Kunming Institute of Botany, CN | FISH |
| | *Cycas panzhiuhuanensis* | Cycadaceae | Panzhihuanenses | **SRR12737664** | Kunming Institute of Botany, CN | GEN |
| | *Cycas pectinata* | Cycadaceae | Indosinensis | **SRR12737663** | Royal Botanical Garden, Kew, UK | GEN |
| | *Cycas revoluta* [c] | Cycadaceae | Asiorientales | ERR845258 | Royal Botanical Garden, Kew, UK | GEN, SB |
| | *Cycas revoluta* [c] | Cycadaceae | Asiorientales | n.a. | Bánovce nad Bebravou, SK | FISH, ICH |
| | *Cycas revoluta* [c] | Cycadaceae | Asiorientales | **SRR12737662** | Kunming Institute of Botany, CN | GEN |
| | *Cycas rumphii* | Cycadaceae | Cycas | **SRR12737661** | Royal Botanical Garden, Kew, UK | GEN |
| | *Cycas sexseminifera* | Cycadaceae | Stangerioides | **SRR12737685** | Kunming Institute of Botany, CN | GEN |
| | *Cycas siamensis* | Cycadaceae | Indosinensis | **SRR12737684** | Royal Botanical Garden, Kew, UK | GEN |
| | *Cycas taitungensis* | Cycadaceae | Asiorientalis | **SRR12737683** | Royal Botanical Garden, Kew, UK | GEN |
| | *Cycas thouarsii* | Cycadaceae | Cycas | **SRR12737682** | Royal Botanical Garden, Kew, UK | GEN |
| subtotal | *19* | | | | | |
| Zamiaceae | *Ceratozamia hildae* | Zamiaceae | n.a. | **SRR12737677** | Royal Botanical Garden, Kew, UK | GEN |
| | *Dioon edule* | Zamiaceae | n.a. | **SRR12737681** | Botanical garden Brno, CZ | GEN, FISH, SB |
| | *Dioon spinulosum* | Zamiaceae | n.a. | **SRR18671860** | Commercial source, CZ | GEN, ICH, SB |
| | *Encephalartos ferox* | Zamiaceae | n.a. | **SRR12737679** | Royal Botanical Garden, Kew, UK | GEN |
| | *Encephalartos manikensis* | Zamiaceae | n.a. | **SRR12737678** | Royal Botanical Garden, Kew, UK | GEN |
| | *Macrozamia communis* | Zamiaceae | n.a. | **SRR18671861** | Commercial source, CZ | GEN, FISH, ICH |
| | *Macrozamia moorei* | Zamiaceae | n.a. | **SRR12737676** | Royal Botanical Garden, Kew, UK | GEN |
| | *Stangeria eriopus* | Zamiaceae | n.a. | **SRR12737680** | Royal Botanical Garden, Kew, UK | GEN |
| | *Zamia fisheri* | Zamiaceae | n.a. | **SRR12737674** | Royal Botanical Garden, Kew, UK | GEN |

| | | | | | | |
|---|---|---|---|---|---|---|
| | *Zamina furfuracea* [d] | Zamiaceae | n.a. | **SRR12737673** | Royal Botanical Garden, Kew, UK | GEN |
| | *Zamina furfuracea* [d] | Zamiaceae | n.a. | n.a. | Botanical garden Brno, CZ | FISH |
| subtotal | *11* | | | | | |
| Other gymnosperms | | | | | | |
| | *Abies sibirica* | Pinaceae | n.a. | ERS243101 | Royal Botanical Garden, Kew, UK | GEN |
| | *Ephedra altissima* | Ephedraceae | n.a. | ERR845261 | Royal Botanical Garden, Kew, UK | GEN |
| | *Ginkgo biloba* | Ginkgoaceae | n.a. | SRR4808604 | NCBI | GEN |
| | *Gnetum gnemon* | Gnetaceae | n.a. | ERR268420 | Royal Botanical Garden, Kew, UK | GEN, SB |
| | *Gnetum montanum* | Gnetaceae | n.a. | SRR4116882 | Fairy Lake BG, Shenzhen, China | GEN |
| | *Juniperus cedrus* | Pinaceae | n.a. | SRR1145775 | NCBI | GEN |
| | *Metasequoia glyptostroboides* | Cupressaceae | n.a. | **SRR12737671** | Royal Botanical Garden, Kew, UK | GEN |
| | *Picea glauca* | Pinaceae | n.a. | ERR1798849 | NCBI | GEN |
| | *Pinus taeda* | Pinaceae | n.a. | SRR1049544 | NCBI | GEN |
| | *Podocarpus macrophyllus* | Podocarpaceae | n.a. | **SRR12737670** | Royal Botanical Garden, Kew, UK | GEN |
| | *Prumnopitys andina* | Podocarpaceae | n.a. | **SRR12737669** | Royal Botanical Garden, Kew, UK | GEN |
| | *Retrophyllum minus* | Podocarpaceae | n.a. | **SRR12737668** | Royal Botanical Garden, Kew, UK | GEN |
| | *Taxus bacata* | Taxaceae | n.a. | ERR268424 | NCBI | GEN |
| | *Welwitschia mirabilis* | Welwitschiaceae | n.a. | ERR845262 | Royal Botanical Garden, Kew, UK | GEN, SB |
| | *Wollemia nobilis* | Araucariaceae | n.a. | **SRR12737672** | Royal Botanical Garden, Kew, UK | GEN |
| subtotal | *15* | | | | | |
| Angiosperms | *Anacyclus pyrethrum* | Asteraceae | n.a. | SRR12002425 | NCBI | GEN |
| | *Anacyclus radiatus* | Asteraceae | n.a. | SRR9822607 | NCBI | GEN |
| | *Amborella trichopoda* | Amborellaceae | n.a. | SRX337118 | NCBI | GEN |
| | *Arabidopsis thaliana* | Brassicaceae | n.a. | ERR031531 | NCBI | GEN |
| | *Arachis hypogaea* | Fabaceae | n.a. | SRR15725034 | NCBI | GEN |
| | *Asparagus officinalis* | Asparagaceae | n.a. | SRR830635 | NCBI | GEN |
| | *Ballantinia antipoda* | Brassicaceae | n.a. | ERR2790837 | NCBI | GEN |
| | *Beta vulgaris* | Amaranthaceae | n.a. | SRR9845562 | NCBI | GEN |
| | *Brassica napus* | Brassicaceae | n.a. | SRR15524610 | NCBI | GEN |
| | *Cardamine amara* | Brassicaceae | n.a. | SRR10230724 | NCBI | GEN |
| | *Cucumis pepo* | Cucurbiteae | n.a. | SRR2531259 | NCBI | GEN |
| | *Fritillaria imperialis* | Liliaceae | n.a. | ERX926174 | NCBI | GEN |
| | *Genlisea nigrocaulis* | Lentibulariaceae | n.a. | SRR1049544 | NCBI | GEN |

| | | | | | | |
|---|---|---|---|---|---|---|
| | *Gossypium hirsutum* | Malvaceae | n.a. | ERR1449079 | NCBI | GEN |
| | *Hordeum vulgare* | Poaceae | n.a. | SRR427194 | NCBI | GEN |
| | *Chenopodium quinoa* | Amaranthaceae | n.a. | DRR057250 | NCBI | GEN |
| | *Lathyrus latifolius* | Fabaceae | n.a. | ERR413120 | NCBI | GEN |
| | *Lilium tsingtauense* | Liliaceae | n.a. | SRR1265940 | NCBI | GEN |
| | *Nicotiana sylvestris* | Solanaceae | n.a. | SRX096295 | NCBI | GEN |
| | *Nicotiana tomentosifomis* | Solanaceae | n.a. | SRR343066 | NCBI | GEN |
| | *Nicotiana tabacum* | Solanaceae | n.a. | SRR343012 | NCBI | GEN, SB |
| | *Oryza sativa* | Poaceae | n.a. | ERR605264 | NCBI | GEN |
| | *Quercus rubor* | Fagaceae | n.a. | SRR2053077 | NCBI | GEN |
| | *Rosa canina* | Rosaceae | n.a. | SRR8265808 | NCBI | GEN |
| | *Secale cereale* | Poaceae | n.a. | ERR505041 | NCBI | GEN |
| | *Tanacetum cinerariifolium* | Asteraceae | n.a. | SRR8627837 | NCBI | GEN |
| | *Tragopogon mirus* [e] | Asteraceae | n.a. | **SRR12710425** | NCBI | GEN |
| | *Vicia faba* | Fabaceae | n.a. | ERR413107 | NCBI | GEN |
| | *Zea mays* | Poaceae | n.a. | SRR512996 | NCBI | GEN |
| subtotal | *29* | | | | | |
| Bryophytes | *Anthoceros punctuatus* | Anthocerotaceae | n.a. | SRR1278954 | NCBI | GEN |
| | *Apopellia endiviifolia* | Pelliaceae | n.a. | SRR8245989 | NCBI | GEN |
| | *Dicranum scoparium* | Dicranaceae | n.a. | SRR8707091 | NCBI | GEN |
| | *Lunularia cruciata* | Lunulariaceae | n.a. | SRR8246012 | NCBI | GEN |
| | *Marchantia polymorpha* | Marchantiaceae | n.a. | SRR3142929 | NCBI | GEN |
| | *Pallavicinia lyellii* | Pallaviciniaceae | n.a. | SRR8245988 | NCBI | GEN |
| | *Physcomitrella patens* | Funariaceae | n.a. | SRR4000524 | NCBI | GEN |
| | *Polytrichum formosum* | Polytrichaceae | n.a. | SRR8707304 | NCBI | GEN |
| subtotal | *8* | | | | | |
| total | *64* | | | | | |

Footnotes

[a] Genomes sequenced within this work are in bold letters. Newly obtained SRAs associate with Bioprojects PRJNA665617 (gymnosperms) and PRJNA634996 (T. mirus).

[b] Type of analysis: GEN - genomic, FISH - telomere FISH, ICH - immunhistochemical labelling, SB - Southern blot hybridization

[c] Three different collections. [d] Two different collections

[e] Originally collected in wild (Palouse, USA) and cultivated in the Brno (CZ) greenhouse

n.a. - not applicable
