# Supplementary material for: Mega‐sized pericentromeric blocks of simple telomeric repeats and their variants reveal patterns of chromosome evolution in ancient Cycadales genomes
Source: Plant J. 2022 Oct 11;112(3):646–63. doi: 10.1111/tpj.15969 (PMC9827991; doi:10.1111/tpj.15969)

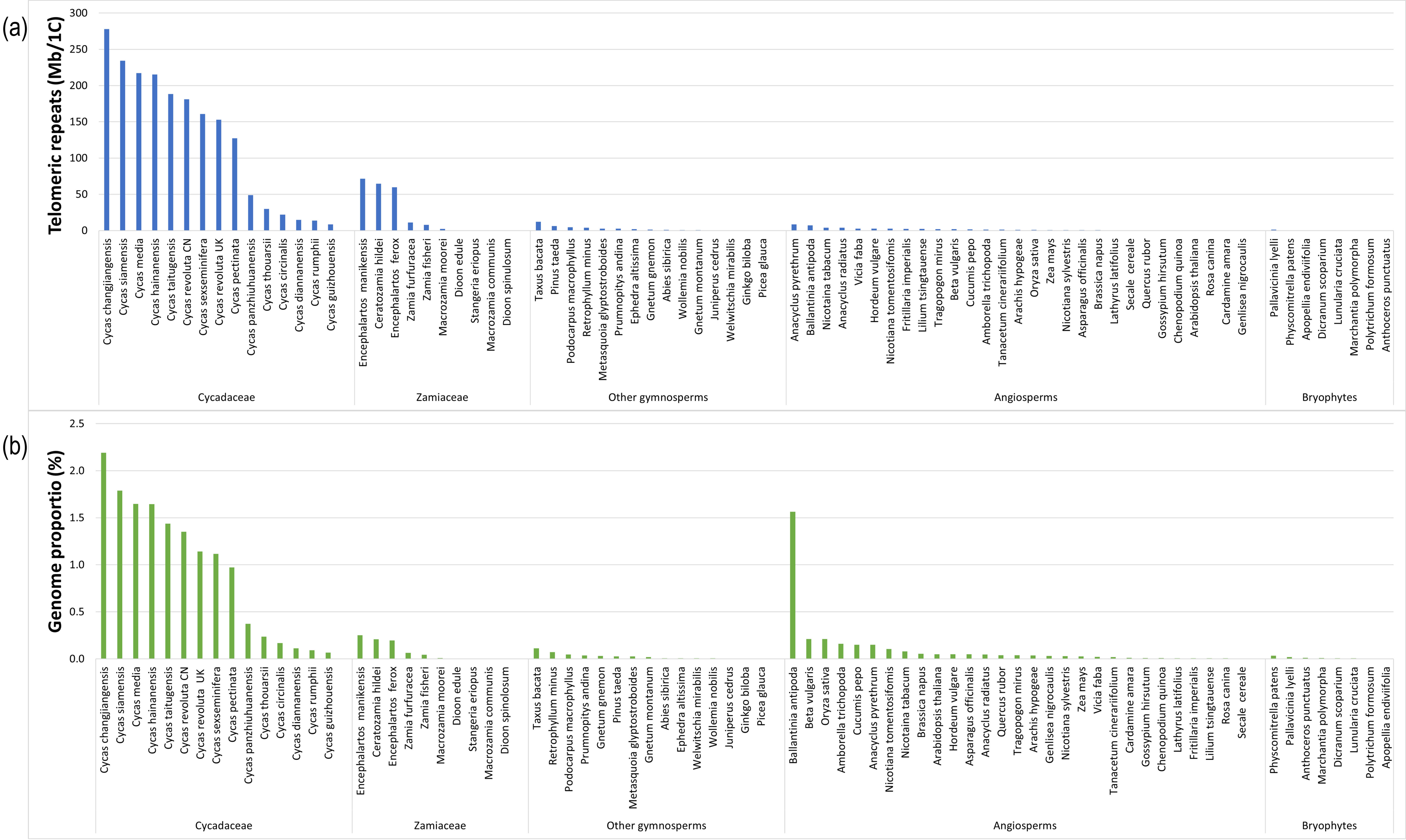

FigureS1. Telomeric repeat abundance in plant genomes expressed in Mb/1C (a) and as a genome proportion in percentage (b). Species in the groups are aligned according to descending abundance.

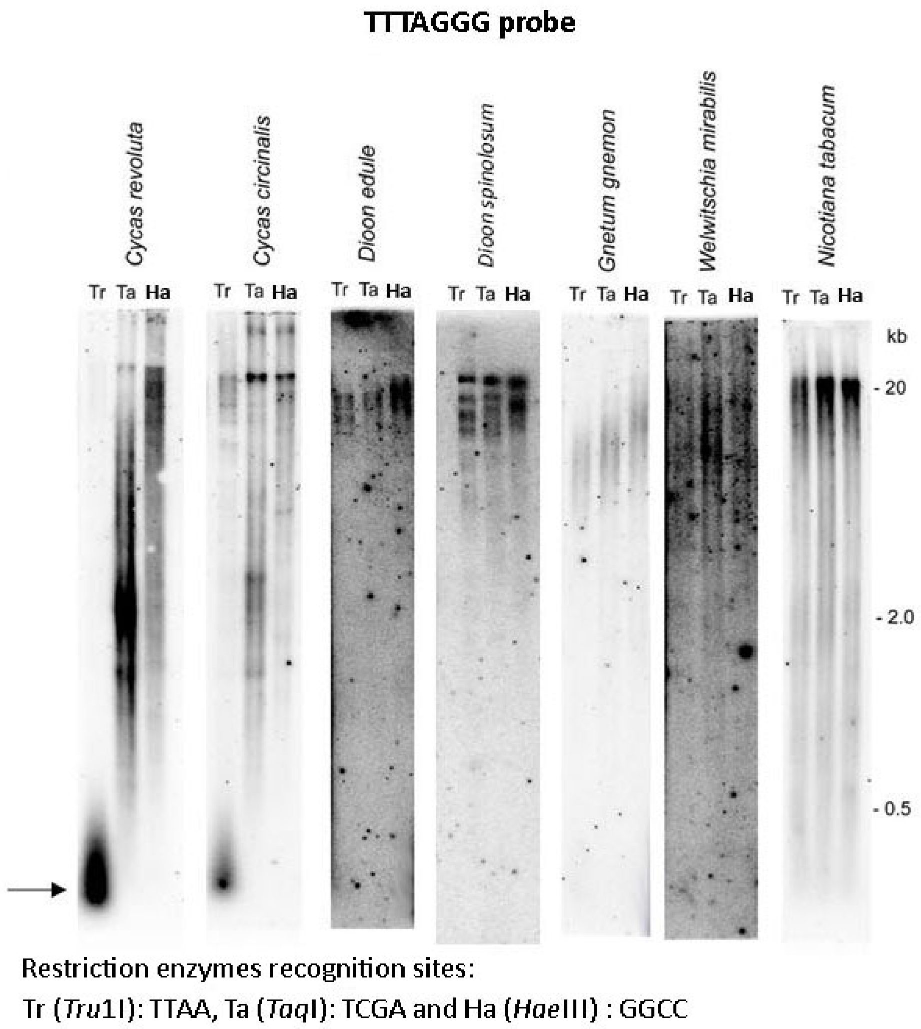

Figure S2. Structural analysis of telomeric repeats in several gymnosperm and angiosperm species by Southern blot hybridization. (a) Terminal telomeric fragments revealed by hybridizing a TTTAGGG probe (inverse complement) to genomic DNAs of six gymnosperms and one angiosperm *(Nicotiana tabacum*) digested with Tru1I (Tr), TaqI (Ta) and HaeIII (Ha) restriction enzymes. Note the extensive digestion with Tru1I of DNAs containing telomeric repeats in *C. revoluta* and *C. circinalis* (arrow).

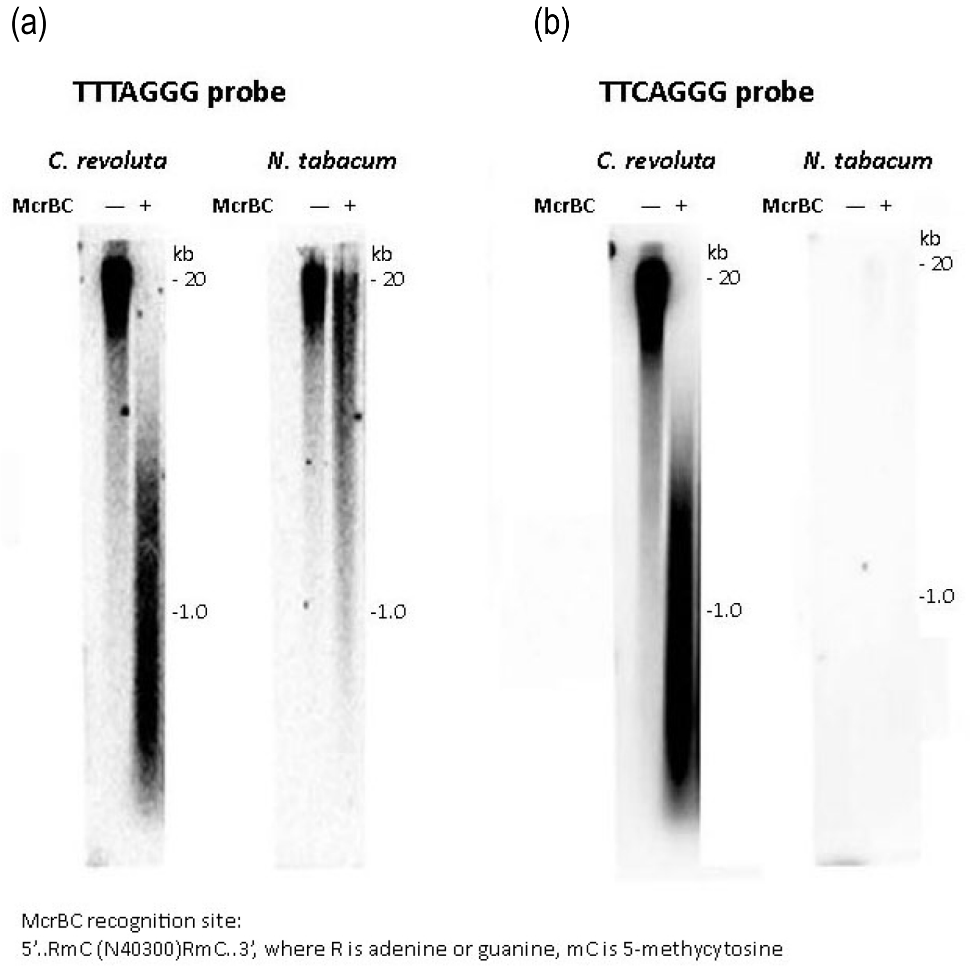

Figure S3. DNA methylation analysis of telomeric repeats by methylation-sensitive restriction enzyme. The genomic DNAs from *C. revoluta* and *N. tabacum* were digested with the McrBC enzyme cutting motifs containing 5-methylcytosine. Hybridization probes were tetrameric oligos composed inverse complements of TTTAGGG (a) and TTCAGGG (b), respectively.

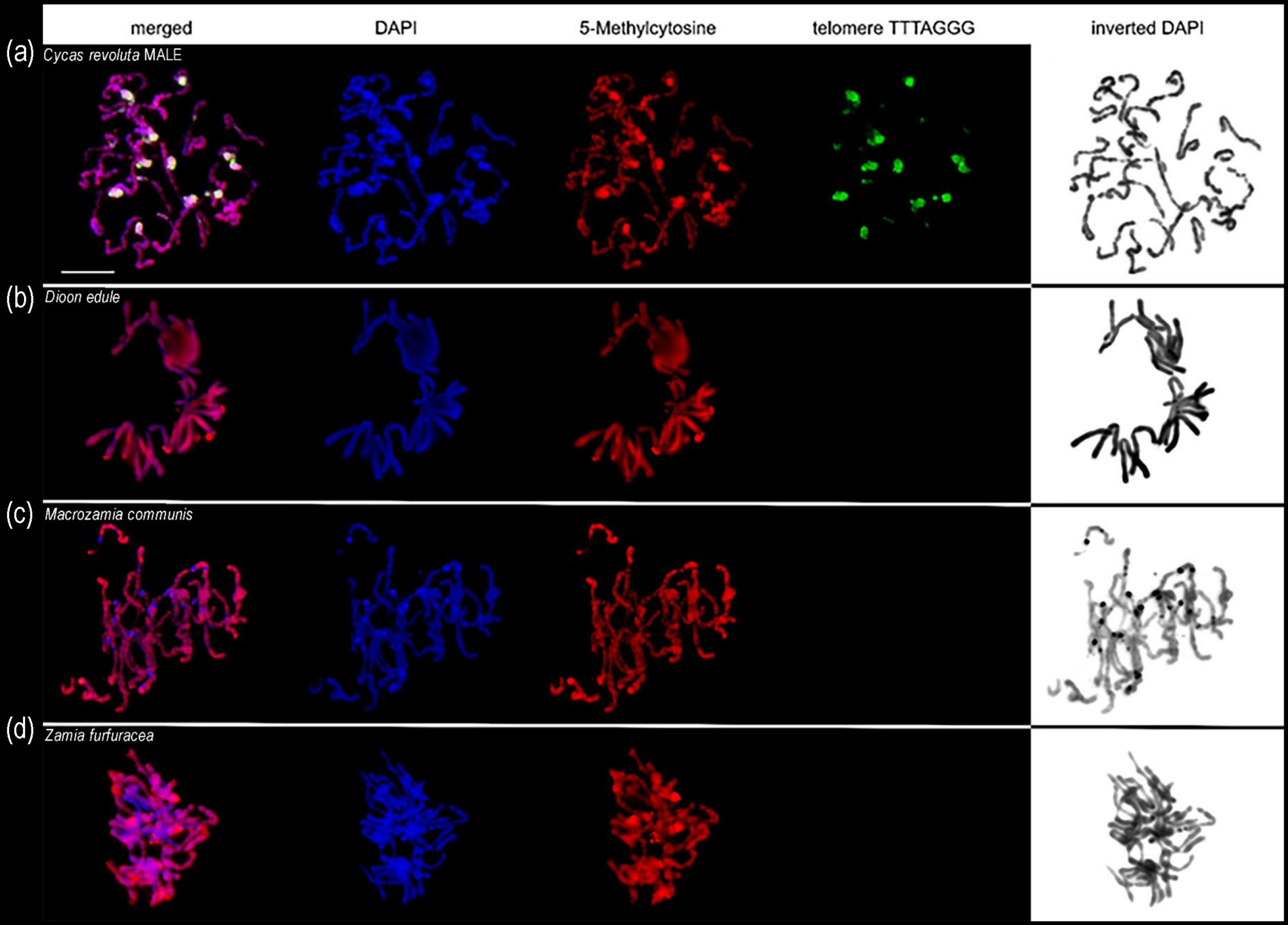

Figure S4. Immunohistochemical staining of cycad prophase chromosomes with an antibody to 5-methylcytosine (5-mC) and by telomere-FISH. Note in *C. revoluta* that there is an accumulation of 5-mC signals overlapping with blocks of telomeric repeats (a). Metaphase, prophase or anaphase chromosomes of *Dioon edule* (b), *Macrozamia communis* (c), and *Zamia furfuracea* (d), respectively, show 5-mC signals with several local maxima and minima. In these three species the telomere-FISH was not provided. Bar = 10 µm.

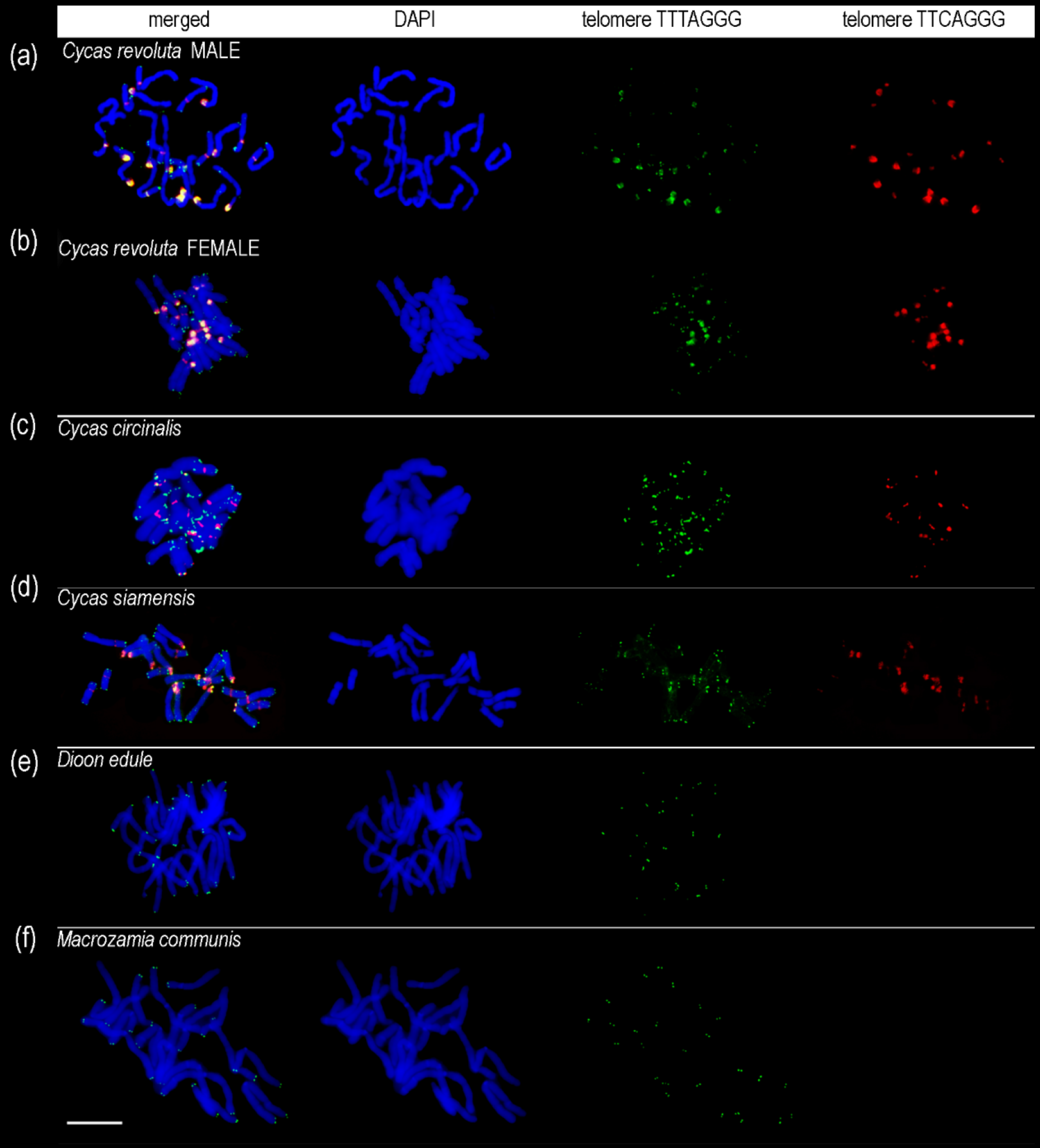

Figure S5. Telomeric FISH showing the location of hybridization signals from two different telomeric probes separately for four species. There were 11 and 12 strong telomeric probe signals in *C. revoluta* male (a) and female (b), respectively. Distribution of telomere probes TTTAGGG (green), and TTCAGGG (red) on chromosomes of *C. circinalis* (c), *C. siamensis* (d), *D. edule* (e) and *M. communis* (f). Bar = 10μm.

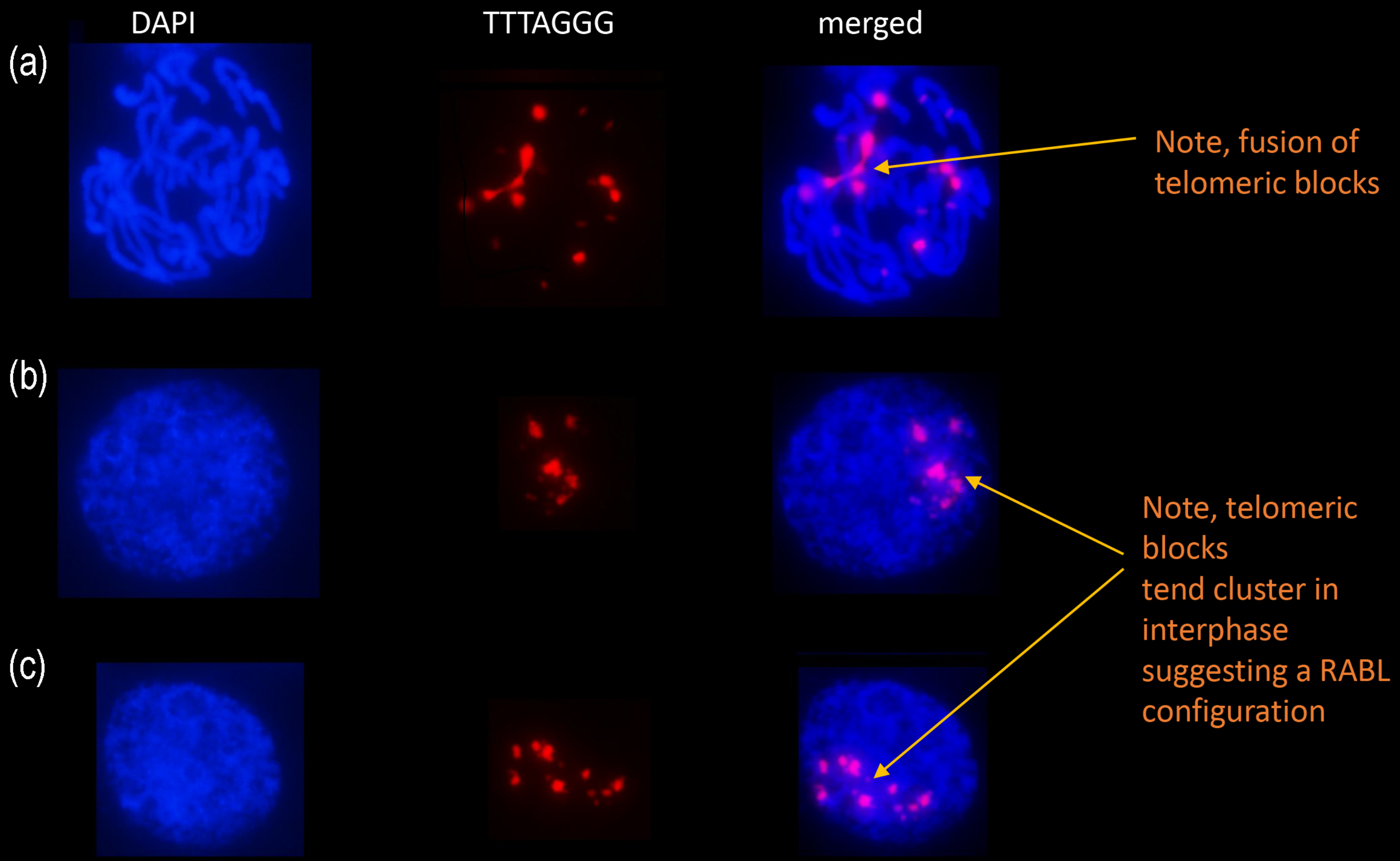

Figure S6. Telomeric FISH of Cycas multipinnata metaphase (a) and interphase (b,c). Note, fusion of telomeric blocks in (a). Note, telomeric blocks tend to cluster in interphase (b, c). Hybridization signals in telomeric positions are barely visible probably due to low copy of repeats and high level of chromosome condensation.

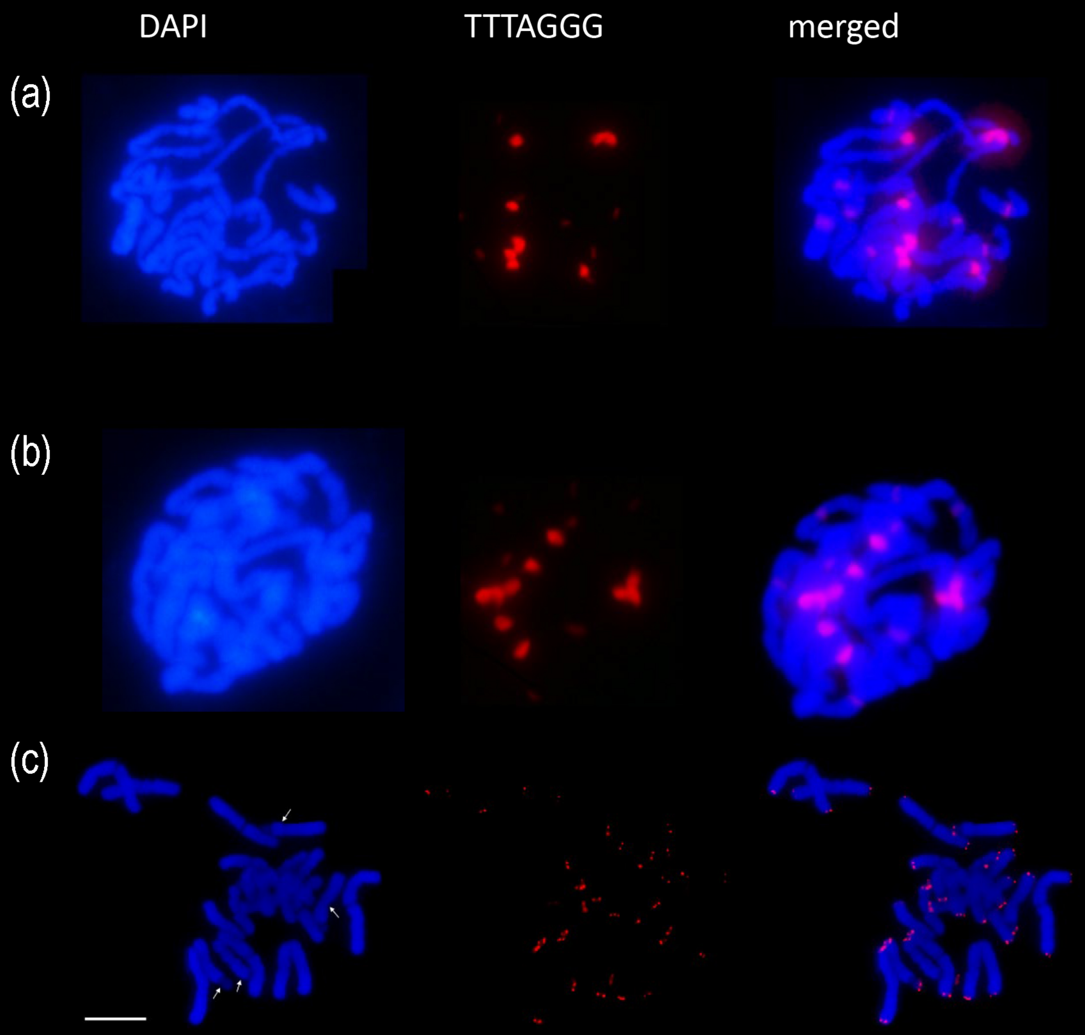

Figure S7. Telomeric FISH of *Cycas debaoensis* (a), *C. multifrondis* (b) and *Z. furfuracea* (c) . In (a,b) note, strong and weak probe hybridization to proximal regions of telocentric and (sub)metacentric chromosomes, respectively. Hybridization signals in telomeric positions are barely visible probably due to low copy of repeats and high level of chromosome condensation. The white arrows point to 4 telocentric chromosomes of *Z. furfuracea* (c), bar = 10µm.

Figure S8. Phylogenetic trees showing very different levels of homogeneity in telomeric arrays between *Cycas revoluta* and *Nicotiana tabacum*. The trees were constructed from 300 aligned sequences. The sequences were obtained by a BLAST search of stand-alone databases of telomeric reads (obtained from mapping files). A 5-mer of the *Arabidopsis* repeat was used as the query.

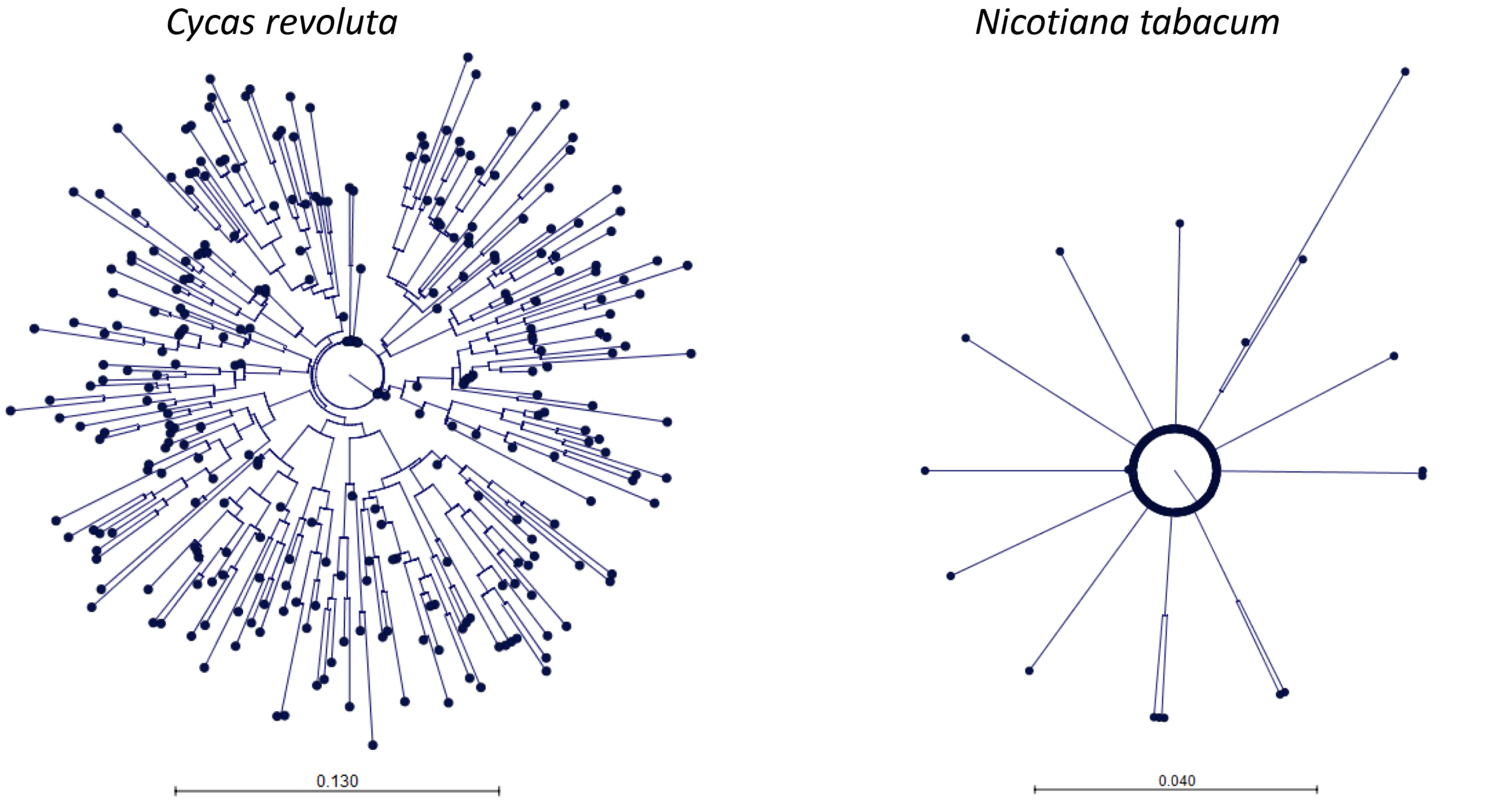

Supplement: Supplementary file 5 — Figure S1. Telomeric repeat abundance in plant genomes expressed in Mb/1C (a) and as a genome proportion in percentages (b). Figure S2. Structural analysis of telomeric repeats in several gymnosperm and angiosperm species by Southern blot hybridization. (a) Terminal telomeric fragments revealed by hybridizing a TTTAGGG probe (inverse complement) to genomic DNAs of six gymnosperms and one angiosperm (Nicotiana tabacum) digested with Tru1I (Tr), TaqI (Ta), and HaeIII (Ha) restriction enzymes. Note the extensive digestion with Tru1I of DNAs containing telomeric repeats in Cycas revoluta and Cycas circinalis (arrow). Figure S3. DNA methylation analysis of telomeric repeats by methylation‐sensitive restriction enzyme. The genomic DNAs from Cycas revoluta and Nicotiana tabacum were digested with the McrBC enzyme cutting motifs containing 5‐mC. Hybridization probes were tetrameric oligos composed inverse complements of TTTAGGG (a) and TTCAGGG (b), respectively. Figure S4. Immunohistochemical staining of cycad prophase chromosomes with an antibody to 5‐methylcytosine (5‐mC) and by telomere‐FISH. Note in Cycas revoluta that there is an accumulation of 5‐mC signals overlapping with blocks of telomeric repeats (a). Metaphase, prophase or anaphase chromosomes of Dioon edule (b), Macrozamia communis (c), and Zamia furfuracea (d), respectively, show 5‐mC signals with several local maxima and minima. In these three species the telomere‐FISH was not provided. Bar = 10 μm. Figure S5. Telomeric FISH showing the location of hybridization signals from two different telomeric probes separately for five cycad species. There were 11 and 12 strong telomeric probe signals in Cycas revoluta male (a) and female (b), respectively. Distribution of telomere probes TTTAGGG (green), and TTCAGGG (red) on chromosomes of Cycas circinalis (c), Cycas siamensis (d), Dioon edule (e) and Macrozamia communis (f). Bar = 10 μm. Figure S6. Telomeric FISH to Cycas multipinnata metaphase (a) and interphase (b,c [file TPJ-112-646-s005.pdf]
