# Supplementary material for: Mega‐sized pericentromeric blocks of simple telomeric repeats and their variants reveal patterns of chromosome evolution in ancient Cycadales genomes
Source: Plant J. 2022 Oct 11;112(3):646–63. doi: 10.1111/tpj.15969 (PMC9827991; doi:10.1111/tpj.15969)

Supplementary Data S1.
Example of randomly selected reads from Cycas revoluta with highlighted telomeric variants.
Reads were obtained by mapping of Illumina genomic reads to the Arabidopsis 14-mer reference sequence.

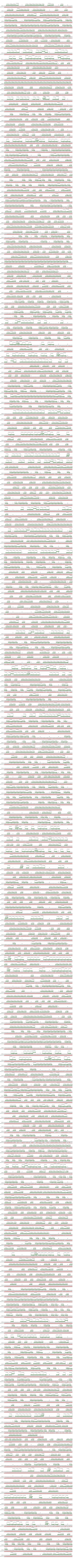

Supplement: Supplementary file 6 — Data S1. Example of randomly selected reads from Cycas revoluta with highlighted telomeric variants. Reads were obtained by mapping of Illumina genomic reads to the Arabidopsis 14‐mer reference sequence. [file TPJ-112-646-s003.pdf]
